# HIV and the Risk of Direct Obstetric Complications: A Systematic Review and Meta-Analysis

**DOI:** 10.1371/journal.pone.0074848

**Published:** 2013-10-04

**Authors:** Clara Calvert, Carine Ronsmans

**Affiliations:** 1 Department of Infectious Disease Epidemiology, London School of Hygiene and Tropical Medicine, London, United Kingdom; University of Cape Town, South Africa

## Abstract

**Background:**

Women of reproductive age in parts of sub-Saharan Africa are faced both with high levels of HIV and the threat of dying from the direct complications of pregnancy. Clinicians practicing in such settings have reported a high incidence of direct obstetric complications among HIV-infected women, but the evidence supporting this is unclear. The aim of this systematic review is to establish whether HIV-infected women are at increased risk of direct obstetric complications.

**Methods and findings:**

Studies comparing the frequency of obstetric haemorrhage, hypertensive disorders of pregnancy, dystocia and intrauterine infections in HIV-infected and uninfected women were identified. Summary estimates of the odds ratio (OR) for the association between HIV and each obstetric complication were calculated through meta-analyses. In total, 44 studies were included providing 66 data sets; 17 on haemorrhage, 19 on hypertensive disorders, five on dystocia and 25 on intrauterine infections. Meta-analysis of the OR from studies including vaginal deliveries indicated that HIV-infected women had over three times the risk of a puerperal sepsis compared with HIV-uninfected women [pooled OR: 3.43, 95% confidence interval (CI): 2.00–5.85]; this figure increased to nearly six amongst studies only including women who delivered by caesarean (pooled OR: 5.81, 95% CI: 2.42–13.97). For other obstetric complications the evidence was weak and inconsistent.

**Conclusions:**

The higher risk of intrauterine infections in HIV-infected pregnant and postpartum women may require targeted strategies involving the prophylactic use of antibiotics during labour. However, as the huge excess of pregnancy-related mortality in HIV-infected women is unlikely to be due to a higher risk of direct obstetric complications, reducing this mortality will require non obstetric interventions involving access to ART in both pregnant and non-pregnant women.

## Introduction

The substantial burden of HIV infection amongst women of reproductive age in sub-Saharan Africa and the maternal health risks that these women are challenged with has lead to HIV and maternal mortality being described as two intersecting epidemics [1], [2]. Many pregnant women in this region face not only the threat of dying from the direct complications of pregnancy and delivery, but also from complications arising from advancing HIV disease. Given this intersection, it is important to understand whether and how HIV interacts with pregnancy.

The biological interaction between HIV and pregnancy is not well understood. It has been argued that pregnancy may accelerate HIV progression as pregnancy is associated with suppressed immune function independent of HIV status [3], [4]. However, the epidemiological evidence supporting this hypothesis is weak. A systematic review investigating the effects of pregnancy on HIV progression and survival found no evidence that pregnancy increased progression to an HIV-related illness or a fall in CD4 count to fewer than 200 cells per cubic millilitre. The same review showed weak evidence that pregnant women were more likely to progress to an AIDS-defining illness or death compared with their non-pregnant counterparts but this was based on only six studies [5].

Clinicians working in settings where HIV is highly prevalent have reported a high incidence of direct obstetric complications in HIV-infected pregnant women [6]. Some researchers have also hypothesised that HIV may increase the risk of direct obstetric complications, though the evidence was based on very few studies with small sample sizes [2], [7]. There are several biological pathways which may explain such an association. Firstly, the compromised immune status and general poor health of HIV-infected women may leave them more vulnerable to infections, including puerperal sepsis [8]. Secondly, it has been suggested that HIV-related thrombocytopenia, where there is a low platelet count in the blood, may increase a woman's risk of haemorrhage [9]. Additionally, social factors such as poor access to healthcare increase a woman's risk of obstetric complications, and may be exacerbated in HIV-infected women due to the discrimination and stigma these women face in some settings [10].

To date there has been no effort to synthesise the empirical evidence on the association between HIV and direct obstetric complications. The aim of this study is to investigate whether HIV increases the risk of obstetric complications, by systematically reviewing literature which compares the risk of obstetric complications in HIV-infected and uninfected women. The obstetric complications which were pre-specified for this review are obstetric haemorrhage, pregnancy-induced hypertension, dystocia and intrauterine infections.

## Methods

### Search Strategy

Pubmed, Embase, Popline and African Index Medicus were searched up to 6^th^ July 2011 using search terms for HIV, pregnancy and the following direct obstetric complications: obstetric haemorrhage, pregnancy-induced hypertension, dystocia and intrauterine infections (see Supplementary File S1 for the full search strategy). There were no language or publication date restrictions. All abstracts were reviewed by a single author (CC) and a 20% sample of abstracts was independently reviewed by a second researcher. Full text copies of potentially relevant papers were obtained and the reference lists of review articles and articles which were included in this systematic review were searched for further relevant publications.

### Eligibility Criteria

Studies were eligible for inclusion if they compared the occurrence of direct obstetric complications during pregnancy, delivery and/or up to 365 days postpartum between HIV-infected and uninfected women using a cohort, cross-sectional or case-control design. Obstetric complications relevant for this review were categorised as: obstetric haemorrhage (including placenta praevia, placental abruption, antepartum haemorrhage, peri- or postpartum haemorrhage and retained placenta); pregnancy-induced hypertension (including eclampsia and pre-eclampsia); dystocia (including prolonged or obstructed labour, abnormal presentation and uterine rupture); and intrauterine infections (including puerperal sepsis, wound infection and endometritis). Studies were required to have a sample size of at least 30 women in each study group with no restrictions on country, dates or whether the study was population or facility based.

### Data Extraction and management

Data were extracted by a single author (CC) on: study location, dates, design and population, definition and ascertainment of the obstetrical outcome (e.g. whether haemorrhage was ascertained through visual estimate or actual measurement of blood loss), the mode of delivery, gestational age at recruitment and length of postpartum follow-up, HIV prevalence in the study population, whether antiretroviral therapy (ART) was available, the number of women with the obstetric complication by HIV status, the type of denominator (pregnancy, live births or women) and the denominator.

Study populations described in more than one paper were included only once, using data from the paper with the most detailed information. When more than one obstetrical outcome was evaluated in a single study, these were extracted and treated as separate data sets.

### Assessment of risk of bias

The risk of bias for each data set was assessed using the component approach adopted by The Cochrane Collaboration [11]. All data sets were assessed on the definition and ascertainment of the obstetric complication, the completeness of data, adjustment for confounding and selection of the comparison group. Each of the quality criteria were classified as having a low risk or high risk of bias for each data set. For example, a data set was classified as having a high risk of bias for outcome ascertainment if methods which were likely to lead to cases being missed were used (e.g. hospital record review). Where there was insufficient information to assess the risk of bias, the data set was classified as at an unclear risk of generating bias.

### Statistical Methods

All analyses were carried out using STATA 12.0. The association between HIV and each obstetric complication was estimated using odds ratios (OR). Summary measures of effect for each obstetric complication were obtained by conducting a random-effects meta-analysis of the best effect estimate available from each study. Where an adjusted OR was available from the paper, this was taken as the best estimate; otherwise the crude estimate was used. Articles do not generally state whether there is overlap between categories of obstetric complications, for example, whether the women who have puerperal sepsis are also the women who are included as having endometritis. We therefore only provide summary estimates for sub-categories within each broad obstetric grouping. As the effect of HIV on the obstetric complications may vary by the mode of delivery, studies which included either vaginal deliveries only or both vaginal and caesarean deliveries were considered separately from studies which only included caesareans. Publication bias was assessed using funnel plots and was formally tested using Begg's test [12].

Additionally, for data sets which included vaginal and caesarean section deliveries, a meta-analysis was conducted to assess whether HIV-infected women had increased odds of caesarean. ORs were computed for each study rather than each data set.

## Results

### Search Strategy Results

**Figure 1:**
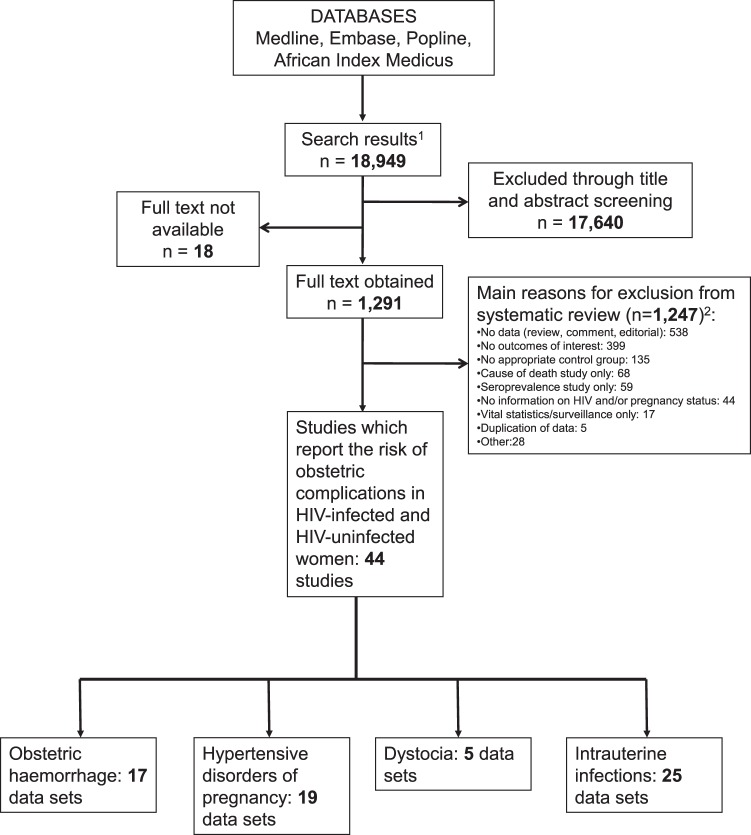
Flow chart of study selection for inclusion in the systematic review. ^1^After removal of duplicates ^2^Articles may have been excluded for multiple reasons.

We initially identified 18,949 titles and abstracts and 1,291 of these were retained for full text review (Figure 1). Of the 1,291 articles, 1,247 were excluded as they did not contain relevant data. A total of 44 studies, providing 66 data sets, were included. Seventeen data sets contained information on obstetric haemorrhage (one caesarean only study), 19 on hypertensive disorders of pregnancy (one caesarean only study), five on dystocia and 25 data sets contained information on intrauterine infections (12 caesarean only studies and one study which was stratified by mode of delivery and therefore provided two data sets).

### Study Characteristics

Table 1 describes the 51 eligible data sets based on vaginal deliveries or all modes of delivery. The 15 data sets which only included women undergoing a caesarean section are described in Table 2. Overall, study populations were from Spain, [13]–[15] France, [16] the UK, [17] Germany, [18] Holland, [19] Italy, [20] the USA, [21]–[27] Mexico, [28] Dominican Republic, [29] Brazil, [30], [31] Kenya, [32]–[35], Ethiopia, [36] Rwanda, [37], [38] Uganda, [39]–[41] Nigeria, [42]–[44] Zimbabwe, [45] South Africa, [46]–[50] India, [51], [52] and Thailand [53], [54]. One study was conducted in Italy, Spain, Sweden, Poland and Ukraine [55] and another was conducted in Malawi, Tanzania and Zambia [56]. All studies were conducted in health facilities. Thirty-four of the data sets (52%) were conducted when ART was available in the study population.

**Table 1:** Summary of studies of HIV and obstetric complications which included births by vaginal delivery.

Reference	Study design		Study Setting	Study Population	Mode of delivery in each study group	ART Available	Definition of obstetric complication	% of HIV positive with obstetric complication *(total number of HIV+ women)*	% of HIV negative with obstetric complication *(total number of HIV+ women)*	Crude Odds Ratio (95% CI)	Adjusted Odds Ratio(95% CI)	
**Haemorrhage**												
**Aboud ** ***et al.*** ** 2009 ** [**56**]	**Prospective Cohort [from a randomised controlled trial (RCT)]**		Multiple hospitals and antenatal clinics in Malawi (Blantyre and Lilongwe), Tanzania (Dar es Salaam) and Zambia (Lusaka) (2001–2003)	All HIV+ women enrolled and one HIV− woman enrolled for every five HIV+ women.	No information provided	No	Antepartum haemorrhage (no further details)	0.5 *(1,558)*	0 *(271)*	2.98 (0.17–51.72)	-	
							Postpartum haemorrhage (no further details)	0.6 *(1,558)*	0 *(271)*	3.68 (0.22–63.02)	-	
**Azira ** ***et al.*** **, 2010 ** [**16**]	**Retrospective Cohort**		One maternity hospital in Paris, France (2001–2006)	All HIV+ women with an undetectable viral load at 36 weeks gestation and one HIV− control for each HIV+ woman matched for parity, previous c-section and geographic origin. Excluded deliveries before 37^th^ week of gestation, multiple pregnancies, non cephalic presentation or elective c-section and for HIV+ women viral load had to be undetectable.	**HIV+**: 17.8% had a c-section **HIV−**: 15.7% had a c-section	Yes	Postpartum haemorrhage defined as blood loss≥500mL after delivery	12.3 *(146)*	18.5 *(146)*	0.62 (0.32–1.18)	-	
**Braddick ** ***et al.,*** ** 1990 ** [**32**]	**Prospective Cohort**		One maternity hospital in Nairobi, Kenya (1986–1989)	All HIV+ women and HIV− women who lived close to the follow-up clinic.	**HIV+**: 0.5% had a c-section **HIV−**: No c-sections	No	Antepartum haemorrhage defined as bleeding during the third trimester	8.1 *(161)*	2.9 *(307)*	2.91 (1.22–6.96)	-	
**Chamiso, 1996 ** [**36**]	**Prospective Cohort**		One maternity hospital in Addis Ababa, Ethiopia (1993–1995)	All HIV+ women and HIV− women matched to the HIV+ women for age and parity.	**HIV+**: 6.5% had a c-section **HIV−**: 9.2% had a c-section	No	Placenta praevia (no further details)	2.2 *(92)*	2.9 *(173)*	0.75 (0.14–3.93)	-	
							Postpartum haemorrhage (no further details)	0 *(92)*	1.2*(173)*	0.37 (0.02–7.81)	-	
							Retained placenta (no further details)	*0 (92)*	1.7 *(173)*	0.26 (0.01–5.15)	-	
**Chanrachakul ** ***et al.,*** ** 2001 ** [**53**]	**Retrospective Cohort**		One tertiary hospital in Bangkok, Thailand (1991–1999)	All nulliparous HIV+ women delivering from 1991–1999 and all non-private, nulliparous HIV− women admitted in 1998. Excluded emergency c-section, deliveries before 37^th^ week of gestation, multiple pregnancies or non cephalic presentation.	**HIV+**: 14.6% had a c-section **HIV−**: 15.0% had a c-section; analysis restricted to vaginal deliveries	No^1^	Postpartum haemorrhage (no further details)	7.3 *(82)*	2.8 *(1,540)*	2.75 (1.13–6.66)	-	
							Retained placenta (no further details)	1.2 *(82)*	0.7 *(1,540)*	1.89 (0.24–14.94)	-	
**De Groot ** ***et al.,*** ** 2003 ** [**46**]	**Retrospective Cohort**		One high risk obstetric unit in Bloemfontein, South Africa (2001)	All HIV+ women and two HIV- controls for every HIV+ woman enrolled.	**HIV+**: 56.8% had a c-section **HIV−**: 55.7% had a c-section	No^1^	Antepartum haemorrhage defined as any bleeding occurring during pregnancy but before delivery	13.6 *(81)*	8.2 *(170)*	1.75 (0.76–4.05)	-	
							Postpartum haemorrhage defined as a fall in Hb level≥3g/dL associated with vaginal bleeding	4.9 *(81)*	6.5 *(170)*	0.75 (0.23–2.43)	-	
**Figueroa-Damian, 1999 ** [**28**]	**Prospective Cohort**		Institute of Perinatology in Mexico City, Mexico (1989–1997)	44 HIV+ women and two HIV− controls for each HIV+ woman, matched on age and socioeconomic status.	**HIV+**: 29.9% had a c-section **HIV−**: 51.2% had a c-section	Yes	Postpartum haemorrhage (no further details)	2–.3 *(44)*	0 *(88)*	6.10 (0.24–152.93)	-	
**Haeri ** ***et al.,*** ** 2009 ** [**21**]	**Retrospective Cohort**		Two tertiary care centres in Columbia and North Carolina, USA (2000–2007)	All HIV+ women on ART and two HIV− controls for each HIV+ woman matched for age, race, parity, care location, delivery mode, insurance type and year of delivery. Excluded deliveries before 20 weeks gestation.	**HIV+**: 51.0% had a c-section **HIV−**: 52.0% had a c-section	Yes	Placental Abruption (no further details)	1.3 *(151)*	1.7 *(302)*	0.80 (0.15–4.16)	-	
**Kourtis ** ***et al.,*** ** 2006 ** [**22**]	**Retrospective Cohort**		20% of all community hospitals in the USA (1994 & 2003)	All HIV+ and HIV− pregnant women between 15-44 years of age who were hospitalised.	No information provided	Yes	Antepartum haemorrhage defined according to ICD-9 codes	2.8 *(12,378)*	1.2 *(8,784,767)*	2.33 (2.09–2.59)	-	
**Leroy ** ***et al*** **., 1998 ** [**37**]	**Prospective Cohort**		One tertiary hospital in Kigali, Rwanda (1992–1993)	All HIV+ women and one HIV− control for each HIV+ woman matched for age. Only included women resident in Kigali who attended antenatal clinic two days a week and wished to deliver in the hospital.	**HIV+**: 5.8% had a c-section **HIV−**: 6.0% had a c-section	No	Postpartum haemorrhage (no further details)	1.4 *(364)*	0 *(365)*	11.18 (0.62–202.99)	-	
							Retained placenta (no further details)	12.1 *(305)*	10.1 *(308)*	1.23 (0.74–2.05)	-	
**Lionel ** ***et al*** **., 2008 ** [**51**]	**Retrospective Cohort**		One hospital in Vellore, India (2000–2002)	All HIV+ and HIV− women.	**HIV+**: 58.7% had a c-section **HIV−**: 21.5% had a c-section	Yes	Major placenta praevia	0.9 (109)	0.5 *(23,277)*	2.06 (0.29–14.92)	-	
							Placental abruption (Grade III)	0.9 (109)	0.1 *(23,277)*	19.58 (2.51–153.02)	-	
							Postpartum haemorrhage (no further details)	0 *(109)*	1.2 *(23,277)*	0.39 (0.02–6.31)	-	
**Louis ** ***et al.,*** ** 2006 ** [**23**]	**Retrospective Cohort**		One tertiary hospital in Detroit, USA (2000–2005)	All HIV+ women and a random selection of HIV− women.	**HIV+**: 39.9% had a c-section **HIV−**: 15.8% had a c-section	Yes	Postpartum haemorrhage (no further details)	1.4 *(148)*	5.3 *(152)*	0.25 (0.05–1.18)	-	
**Minkoff ** ***et al.,*** ** 1990 ** [**24**]	**Prospective Cohort**		Four prenatal clinics in New York, USA (1985–1989)	All HIV+ women who had a live, singleton birth; in three of the prenatal clinics all HIV− women were also recruited, and in one of the clinics two HIV− controls were selected for each HIV+ woman.	**HIV+**: 12.0% had a c-section **HIV−**: 18.0% had a c-section	No	Placenta praevia (no further details)	1.2 *(85)*	1.7 *(118)*	0.69 (0.06–7.74)	-	
							Placental abruption (no further details)	0 *(85)*	2.5 *(118)*	0.18 (0.01–3.62)	-	
							Peripartum haemorrhage (no further details)	4.5 *(89)*	4.0 *(126)*	1.14 (0.30–4.37)	-	
							Retained placenta (no further details)	3.4 *(89)*	0.8 *(126)*	4.36 (0.45–42.62)	-	
**Mmiro ** ***et al*** **., 1993 ** [**39**]	**Prospective Cohort**		One university hospital in Kampala, Uganda (1988–1990)	All HIV+ women and a random 10% sample of HIV− women. Only included women who lived within 15km of Mulago and agreed to deliver in the hospital.	No difference in the mode of delivery in HIV+ and HIV− women	No	Antepartum haemorrhage (no further details)	0.9 *(539)*	1.4 *(660)*	0.68 (0.23–2.03)	–	
							Postpartum haemorrhage (no further details)	0.6 *(539)*	0.9 *(660)*	0.61 (0.15–2.45)	-	
**Peret ** ***et al*** **., 1993 ** [**30**]	**Prospective Cohort**		One maternity hospital in Belo Horizonte, Brazil (2001–2003)	82 HIV+ women and 123 HIV− women matched on mode of delivery, gestational age at delivery and maternal age. Only included women if they did not have chronic diseases and/or complications of pregnancy.	**HIV+**: 72.0% had a c-section **HIV−**: 72.0% had a c-section	Yes	Postpartum haemorrhage defined by clinical observation and/or need for at least one of the following interventions: uterotonic drugs, revision of the uterine cavity and the birth canal or curettage	2.4 *(82)*	0 *(123)*	7.67 (0.36–161.86)	-	
**Van Eijk ** ***et al.*** **, 2007 ** [**33**]	**Prospective Cohort**		One large hospital in Kisumu, Kenya (1996–2000)	All women who delivered at the hospital if they had an uncomplicated singleton pregnancy at more than 32 weeks gestation, resided in Kisumu and had no underlying chronic illnesses.	**HIV+**: 3.1% had a c-section **HIV−**: 3.6% had a c-section	Not clear	Peripartum haemorrhage (no further details)	1.4 *(743)*	0.3 *(2,365)*	4.02 (1.58–2.33)	-	
**Hypertensive diseases of pregnancy**												
**Aboud ** ***et al.*** ** 2009 ** [**56**]	**Prospective Cohort (from an RCT)**		Multiple hospitals and antenatal clinics in Malawi (Blantyre and Lilongwe), Tanzania (Dar es Salaam) and Zambia (Lusaka) (2001–2003)	All HIV+ women enrolled and one HIV− woman enrolled for every five HIV+ women.	No information provided	No	Hypertension, with or without proteinuria, measured in the intrapartum period	1.7 *(1,558)*	1.1 *(271)*	1.52 (0.46–5.04)	-	
**Bodkin ** ***et al.,*** ** 2005 ** [**47**]	**Retrospective cohort**		One tertiary hospital in Gautang, South Africa (2003)	A sample of HIV+ women selected using stratified random sampling (stratifying on normal risk, moderate risk or high risk pregnancy) and one HIV− control selected for every two HIV+ women.	Only follows up women in ante-partum period	Yes	Pregnancy-induced hypertension (no further details)	17.0 *(212)*	9.9 *(101)*	1.86 (0.88–3.92)	-	
							Eclampsia ( no further details)	2.8 *(212)*	1.0 *(101)*	2.91 (0.35–24.52)	-	
**Boer ** ***et al.,*** ** 2006 ** [**19**]	**Retrospective cohort**		Two medical centres in Amsterdam and Rotterdam, Holland (1997–2003)	All HIV+ treated with ART and two HIV− controls for each HIV+ woman matched on maternal age, parity, ethnicity, and being singleton or twin. The controls had to be healthy and not referred, not have had obstetric complications in the past and live near the hospital.	**HIV+**: 40.8% had a c-section **HIV−**: 12.8% had a c-section	Yes	Pre-eclampsia (during pregnancy until seven days postpartum) defined according to the definition of the International Society for the Studies of Hypertension in Pregnancy	2.0 *(98)*	1.0 *(196)*	2.02 (0.28–14.57)	-	
**Chamiso, 1996 ** [**36**]	**Prospective Cohort**		One maternity hospital in Addis Ababa, Ethiopia (1993–1995)	All HIV+ women and HIV− women matched to the HIV+ women for age and parity.	**HIV+**: 6.5% had a c-section **HIV−**: 9.2% had a c-section	No	Pregnancy-induced hypertension defined as an increment in systolic blood pressure of 30 mmHg and in diastolic blood pressure of 15 mmHg from the pre- or early pregnancy level of blood pressure	6.5 *(92)*	2.3 *(173)*	2.95 (0.81–10.72)	-	
**De Groot ** ***et al.*** **, 2003 ** [**46**]	**Retrospective Cohort**		One high risk obstetric unit in Bloemfontein, South Africa (2001)	All HIV+ women and two HIV− controls for every HIV+ woman enrolled.	**HIV+**: 56.8% had a c-section **HIV−**: 55.7% had a c-section	No^1^	Pre-eclampsia defined as systolic blood pressure of ≥140 mm Hg or a diastolic blood pressure of ≥ 90 mmHg, on at least 2 occasions 4 hours or more apart and proteinuria of ≥ 0.3 g/24 hours)	43.2 *(81)*	35.9 *(170)*	1.36 (0.79–2.33)	-	
							Eclampsia defined as 1+ convulsions which could not be explained by other cerebral conditions, in a patient with pre-eclampsia	7.4 *(81)*	17.1 *(170)*	0.39 (0.15–0.98)	-	
**Figueroa-Damian, 1999 ** [**28**]	**Prospective Cohort**		Institute of Perinatology in Mexico City, Mexico (1989–1997)	44 HIV+ women and two HIV− controls for each HIV+ woman, matched on age and socioeconomic status.	**HIV+**: 29.9% had a c-section **HIV−**: 51.2% had a c-section	Yes	Acute hypertensive disorder of pregnancy (no further details)	2.3 *(44)*	4.6 *(88)*	0.49 (0.05–4.51)	-	
**Frank ** ***et al.,*** ** 2004 ** [**48**]	**Retrospective Cohort**		One tertiary hospital and five primary care clinics in Johannesburg, South Africa (2002)	Random sample of HIV+ and HIV− pregnant Soweto residents who delivered at a gestational age of 20 weeks or more in a public health facility.	No information provided	No	Pregnancy-induced hypertension which includes proteinuric hypertension, gestational hypertension, non proteinuric hypertension and chronic hypertension	14.9 *(704)*	14.8 *(1,896)*	1.01 (0.79–1.28)	-	
							Pre-eclampsia defined as hypertension (diastolic blood pressure of ≥ 90 mm Hg on 2+ occasions, 4 hours apart) associated with proteinuria which developed after 20 weeks of pregnancy	2.1 *(704)*	3.0 *(1,896)*	0.97 (0.59–1.62)	-	
							Eclampsia (no further details)	0.3 *(704)*	0.3 *(1,896)*	0.90 (0.18–4.46)	-	
**Haeri ** ***et al.,*** ** 2009 ** [**21**]	**Retrospective Cohort**		Two tertiary care centres in Columbia and North Carolina, USA (2000–2007)	All HIV+ women on ART and two HIV− controls for each HIV+ woman matched for age, race, parity, care location, delivery mode, insurance type and year of delivery. Excluded deliveries before 20 weeks gestation.	**HIV+**: 51.0% had a c-section **HIV−**: 52.0% had a c-section	Yes	Gestational hypertension (no further details)	0.7 *(151)*	4.3 *(302)*	0.15 (0.02–1.14)	0.18 (0.02–1.40) 2	
							Pre-eclampsia defined according to the national working group for Hypertension in Pregnancy Guidelines	6.0 *(151)*	11.9 *(302)*	0.50 (0.25–1.01)	0.55 (0.26–1.18) 2	
**Kourtis ** ***et al.,*** ** 2006 ** [**22**]	**Retrospective Cohort**		20% of all community hospitals in the USA (1994 & 2003)	All HIV+ and HIV− pregnant women between 15-44 years of age who were hospitalised.	No information provided	Yes	Pre-eclampsia/hypertensive disorders of pregnancy defined according to ICD-9 codes	7.7 *(12,378)*	7.1 *(8,784,767)*	1.09 (1.02–1.17)	-	
**Lionel ** ***et al.*** **, 2008 ** [**51**]	**Retrospective Cohort**		One hospital in Vellore, India (2000–2002)	All HIV+ and HIV− women.	**HIV+**: 58.7% had a c-section **HIV−**: 21.5% had a c-section	Yes	Pregnancy-induced hypertension (no further details)	21.1 *(109)*	8.1 *(23,277)*	3.02 (1.90–4.80)	-	
							Eclampsia (includes antepartum, intrapartum and postpartum)	23.9 *(109)*	0.8 *(23,277)*	38.47 (24.21–61.14)	-	
**Mattar ** ***et al*** **., 2004 ** [**31**]	**Retrospective Cohort**		One obstetric outpatient clinic in Sao Paulo, Brazil (2000–2002)	All women referred to the outpatient obstetric unit. Excluded women with pre-existing hypertension.	No information provided	Yes	Pre-eclampsia defined as hypertension (> = 140 mmHg x 90 mmHg) and proteinuria (> = 300 mg/24h) after 20 weeks of pregnancy	0.8 *(123)*	10.7 *(1,708)*	0.07 (0.01–0.49)	-	
**Mmiro ** ***et al*** **., 1993 ** [**39**]	**Prospective Cohort**		One university hospital in Kampala, Uganda (1988–1990)	All HIV+ women and a random 10% sample of HIV− women. Only included women who lived within 15km of Mulago and agreed to deliver in the hospital.	No difference in the mode of delivery in HIV+ and HIV− women	No	Hypertension defined as diastolic blood pressure >90 mmHg	3.7 *(539)*	6.2 *(660)*	0.58 (0.34–1.01)	-	
**Olagbuji ** ***et al*** **., 2010 ** [**42**]	**Prospective Cohort**		One tertiary hospital in Benin City, Nigeria (2007–2008)	HIV+ women who did not have AIDS, chronic medical disorders predating the pregnancy, multiple gestation or duration of ART intake of less than 8 weeks. A single HIV− control was selected for each HIV+ woman.	**HIV+**: 29.1% had a c-section **HIV−**: 20.2% had a c-section	Yes	Pregnancy-induced hypertension (no further details)	4.9 *(203)*	3.0 *(203)*	1.70 (0.61–4.77)	-	
**Roman-Poueriet ** ***et al*** **., 2009 ** [**29**]	**Retrospective Cohort**		All main obstetric facilities, a social security hospital and two private clinics in La Romana, Dominican Republic (2003–2006)	All HIV+ and HIV− women.	**HIV+**: 42.5% had a c-section **HIV−**: 17.4% had a c-section	Yes	Pregnancy-induced hypertension (no further details)	2.8 *(252)*	0.5 *(9,003)*	5.69 (2.54–12.74)	-	
**Singh ** ***et al.,*** ** 2009 ** [**52**]	**Prospective Cohort**		One hospital in Imphal, India (2006–2008)	50 HIV+ and 100 HIV− women who did not have medical or obstetric complications during pregnancy.	**HIV+**: 32.0% had a c-section **HIV−**: 10.0% had a c-section	Yes	Pre-eclamptic toxaemia (no further details)	6.0 *(50)*	8.0 *(100)*	0.73 (0.19–2.90)	-	
**Suy ** ***et al.,*** ** 2006 ** [**13**]	**Prospective Cohort**		One referral centre in Barcelona, Spain (2001–2003)	All HIV+ and HIV− women delivering after at least 22 weeks of pregnancy.	No information provided	Yes	Pre-eclampsia defined as the new onset of hypertension with 2 readings ≥6 hours apart of more than 140 mmHg systolic during gestation, delivery or immediate postpartum period, plus a dipstick reading of at least 1+ for proteinuria (0.1 g/l) confirmed by > 300 mg/24 h urine collection after 22 weeks of pregnancy	11.0 *(82)*	2.9 *(8,768)*	4.18 (2.07–8.46)	-	
**Waweru ** ***et al.*** **, 2009 ** [**34**]	**Prospective Cohort**		One maternity hospital in Kenya, Nairobi (Study dates not provided)	57 HIV+ and HIV− women who were randomly selected.	Only follows up women in ante-partum period	Not clear	Pre-eclampsia (no further details provided)	17.5 *(57)*	12.3 *(57)*	1.52 (0.53–4.32)	-	
**Wimalasundera ** ***et al.*** **, 2002 ** [**17**]	**Prospective Cohort**		Two hospitals in London, UK (1990–2001)	214 HIV+ women and a single HIV− control for each HIV+ woman matched for age, parity and ethnic origin.	Only follows up women in ante-partum period	Yes	Pre-eclampsia defined according to Higgins and de Swiet [74]	4.2 *(214)*	5.6 *(214)*	0.74 (0.30–1.79)	-	
**Dystocia**												
**Chanrachakul ** ***et al.,*** ** 2001 ** [**53**]		Retrospective Cohort	One tertiary hospital in Bangkok, Thailand (1991–1999)	All nulliparous HIV+ women delivering from 1991–1999 and all non-private, nulliparous HIV− women admitted in 1998. Excluded emergency c-section, deliveries before 37^th^ week of gestation, multiple pregnancies or non cephalic presentation.	**HIV+**: 14.6% had a c-section **HIV−**: 15.0% had a c-section; analysis restricted to vaginal deliveries	No^1^	Prolonged labour defined as labour longer than 12 hours	29.2 *(82)*	5.0 *(1,540)*	7.86 (4.64–13.33)	-	
**Leroy ** ***et al*** **., 1998 ** [**37**]		Prospective Cohort	One tertiary hospital in Kigali, Rwanda (1992–1993)	All HIV+ women and one HIV− control for each HIV+ woman matched for age. Only included women resident in Kigali who attended antenatal clinic two days a week and wished to deliver in the hospital.	**HIV+**: 5.8% had a c-section **HIV−**: 6.0% had a c-section	No	Dystocia (no further details)	7.7 *(349)*	7.5 *(349)*	1.04 (0.59–1.82)	-	
							Abnormal presentation (no further details)	7.0 *(356)*	5.6 *(360)*	1.28 (0.70–2.36)	-	
**Lionel ** ***et al.*** **, 2008 ** [**51**]		Retrospective Cohort	One hospital in Vellore, India (2000–2002)	All HIV+ and HIV− women.	**HIV+**: 58.7% had a c-section **HIV−**: 21.5% had a c-section	Yes	Uterine Rupture (no further details)	0.9 *(109)*	0.3 *(23,277)*	2.75 (0.38–19.98)	-	
**Minkoff ** ***et al.,*** ** 1990 ** [**24**]		Prospective Cohort	Four prenatal clinics in New York, USA (1985–1989)	All HIV+ women who had a live, singleton birth; in three of the prenatal clinics all HIV− women were also recruited, and in one of the clinics two HIV− controls were selected for each HIV+ woman.	**HIV+**: 12.0% had a c-section **HIV−**: 18.0% had a c-section	No	Abnormal presentation (no further details)	4.8 *(84)*	5.9 *(118)*	0.79 (0.22–2.80)	-	
**Wandabwa ** ***et al.,*** ** 2008 ** [**40**]		Case-control	One hospital in Mulago, Uganada (2001–2002)	Case of ruptured uterus and controls were selected from women who had a gestation of 24 or more weeks, delivered a live, singleton baby by vaginal delivery, did not have episiotomy, tear of more than first degree or excessive blood loss.	All vaginal deliveries	No	Uterine rupture diagnosed both by clinical examination and at laparatomy	-	-	2.40 (1.10–4.20)	3.20 (1.50–7.20)^3^	
**Intrauterine infections**												
**Aboud ** ***et al.*** ** 2009 ** [**56**]		Prospective Cohort (from an RCT)	Multiple hospitals and antenatal clinics in Malawi (Blantyre and Lilongwe), Tanzania (Dar es Salaam) and Zambia (Lusaka) (2001–2003)	All HIV+ women enrolled and one HIV− woman enrolled for every five HIV+ women.	No information provided	No	Puerperal sepsis (no further details)	1.8 *(1,558)*	0 *(271)*	10.11 (0.62–166.11)	-	
**Chamiso, 1996 ** [**36**]		Prospective Cohort	One maternity hospital in Addis Ababa, Ethiopia (1993–1995)	All HIV+ women and HIV− women matched to the HIV+ women for age and parity.	**HIV+**: 6.5% had a c-section **HIV−**: 9.2% had a c-section	No	Endometritis (no further details)	9.8 *(92)*	0 *(173)*	39.48 (2.27–686.46)	-	
**Chanrachakul ** ***et al.,*** ** 2001 ** [**53**]		Retrospective Cohort	One tertiary hospital in Bangkok, Thailand (1991–1999)	All nulliparous HIV+ women delivering from 1991–1999 and all non-private, nulliparous HIV− women admitted in 1998. Excluded emergency c-section, deliveries before 37^th^ week of gestation, multiple pregnancies or non cephalic presentation.	**HIV+**: 14.6% had a c-section **HIV−**: 15.0% had a c-section	No^1^	Puerperal infection (no further details)	1.0 *(96)*	1.0 *(1,856)*	1.02 (0.13–7.68)	-	
**Figueroa-Damian, 1999 ** [**28**]		Prospective Cohort	Institute of Perinatology in Mexico City, Mexico(1989–1997)	44 HIV+ women and two HIV− controls for each HIV+ woman, matched on age and socioeconomic status.	**HIV+**: 29.9% had a c-section **HIV−**: 51.2% had a c-section	Yes	Endometritis (no further details)	0*(44)*	4.6*(88)*	0.21 (0.01–4.01)	-	
**Fiore ** ***et al.,*** ** 2004 ** [**55**]		Prospective Cohort	14 references centres in Italy, Spain, Sweden, Poland and Ukraine (1992–2003)	HIV+ women were matched the first HIV− woman delivering after the infected index case on age ethnicity, parity and whether admitted to the delivery unit in active labour.	All vaginal deliveries	Yes	Endometritis (no further details)	4.4 *(250)*	2.0 *(250)*	2.26 (0.77–6.59)	-	
**Kourtis ** ***et al.,*** ** 2006 ** [**22**]		Retrospective Cohort	20% of all community hospitals in the USA (1994 & 2003)	All HIV+ and HIV− pregnant women between 15–44 years of age who were hospitalised.	No information provided	Yes	Major puerperal sepsis identified using ICD-9 codes	2.9 *(12,378)*	0.9 *(8,784,767)*	3.37 (3.03–3.74)767)n sepsis (no further details)ere sleHIV+ woman there were two HIV−	-	
**Lepage ** ***et al.*** ** 1991 ** [**38**]		Prospective Cohort	One hospital in Kigali, Rwanda (1988–1989)	All HIV+ women and an equal number of HIV− women matched for age and parity. Women had to have lived for at least six months in a district within a diameter of <10 Km from the hospital and delivered a live newborn.	No information provided	No	Endometritis (no further details)	0.9 *(215)*	0.9 *(216)*	1.00 (0.14–7.20)	-	
**Minkoff ** ***et al.,*** ** 1990 ** [**24**]		Prospective Cohort	Four prenatal clinics in New York, USA (1985–1989)	All HIV+ women who had a live, singleton birth; in three of the prenatal clinics all HIV− women were also recruited, and in one of the clinics two HIV− controls were selected for each HIV+ woman.	**HIV+**: 12.0% had a c-section **HIV−**: 18.0% had a c-section	No	Endometritis (no further details)	4.4 *(91)*	2.4 *(126)*	1.89 (0.41–8.64)	-	
**Okong ** ***et al.*** ** 2004 ** [**41**]		Case-control	One hospital in Kampala, Uganda (1996–1997)	For each case of postpartum endometritis and myometritis (PPEM), two controls without PPEM were randomly recruited, matched for mode of delivery.	Cases and controls were matched for mode of delivery	No	Endometritis defined as auxiliary temperature ≥38^o^C on 2 different occasions 24 hours apart, with a tender uterus and foul-smelling or purulent vaginal discharge between delivery and 42 days postpartum	-	-	2.74 (1.34–5.65)	-	
**Onah ** ***et al.*** **, 2007 ** [**43**]		Retrospective Cohort	One university hospital in Enugu, Nigeria (2002–2004)	All HIV+ women and for every HIV+ woman the next two HIV− women who delivered were selected as controls.	**HIV+**: 8.1% had a c-section **HIV−**: 11.0% had a c-section	Yes	Puerperal sepsis (no further details)	8.1 *(62)*	0 *(100)*	19.23 (1.04–354.04)	-	
**Peret ** ***et al*** **., 1993 ** [**30**]		Prospective Cohort	One maternity hospital in Belo Horizonte, Brazil (2001–2003)	82 HIV+ women and 123 HIV− women matched for mode of delivery, gestational age at delivery and maternal age. Only included women if they did not have chronic diseases and/or complications of pregnancy.	**HIV+**: 72.0% had a c-section **HIV−**: 72.0% had a c-section	Yes	Endometritis defined as febrile morbidity with a tender uterus and/or foul-smelling vaginal discharge	4.9 *(82)*	0 *(123)*	14.16 (0.75–266.61)	-	
**Temmerman ** ***et al.,*** ** 1994 ** [**35**]		Prospective Cohort	One health centre in Nairobi, Kenya (1989–1991)	All HIV+ women and a sample of HIV− women matched for age and parity to each HIV+ woman.	**HIV+**: 1.9% had a c-section **HIV−**: 3.8% had a c-section	No	Postpartum endometritis diagnosed if at ≥two of the following symptoms were present: fever of >38^o^C, foul lochia and uterine tenderness	10.3 *(253)*	4.2 *(265)*	2.64 (1.28–5.47)	-	

1 Information was not supplied in the published paper so whether antiretroviral treatment should have been available was based on the study dates and study location; for two studies it was not clear from the study dates and location whether ART would be available so the information was inferred from the literature.

a) Bangkok, Thailand between 2001: No ART treatment based on the UNAIDS data accessed on 29th October 2012 at http://www.unaids.org/en/dataanalysis/datatools/aidsinfo/.

b) Bloemfontein, South Africa in 2001: No ART treatment based on the UNAIDS data accessed on 29th October 2012 at http://www.unaids.org/en/regionscountries/countries/southafrica/.

2 Adjusted for smoking and cocaine use.

3 Adjusted for age, type of house, the distance from home to Mulago hospital, permission to attend health unit, person paying for hospital upkeep, previous length of labour and previous delivery by c-section.

**Table 2:** Summary of studies of HIV and obstetric complications which only looked at births by caesarean section.

Reference	Study design	Study Setting	Study Population	ART Available	Definition of obstetric complication	% of HIV positive with obstetric complication *(total number of HIV+ women)*	% of HIV negative with obstetric complication *(total number of HIV+ women)*	Crude Odds Ratio (95% CI)	Adjusted Odds Ratio (95% CI)
**Haemorrhage**									
**Chama and Morrupa, 2008 ** [**44**]	Prospective Cohort	One university hospital in Maiduguri, Nigeria (2006)	All HIV+ women and an equivalent number of HIV− women who delivered by elective c-section.	Yes	Intra-operative haemorrhage defined as any bleeding during surgery requiring blood transfusion or a fall in packed cell volume ≥4%	23.1 *(52)*	40.4 *(52)*	0.44 (0.19–1.04)	-
**Hypertensive diseases of pregnancy**									
**Chama and Morrupa, 2008 ** [**44**]	Prospective Cohort	One university hospital in Maiduguri, Nigeria (2006)	All HIV+ women and an equivalent number of HIV− women who delivered by elective c-section.	Yes	Postpartum pregnancy-induced hypertension (no further details)	0 *(52)*	1.9 *(52)*	0.33 (0.01–8.21)	-
Sepsis									
**Cavasin ** ***et al.,*** ** 2009 ** [**25**]	Retrospective Cohort	Two health centres (one of which is part of a university hospital) in New Orleans in the USA (1998–2004)	All HIV+ women undergoing c-section; HIV− women were those who delivered by c-section during the same time period.	Yes	Post-operative endometritis defined as temperature elevation above 38°C with uterine tenderness and requiring antibiotics treatment in the absence of other aetiology for fever	12.6 *(119)*	12.1 *(264)*	1.05 (0.54–2.01)	-
**Chama and Morrupa, 2008 ** [**44**]	Prospective Cohort	One university hospital in Maiduguri, Nigeria (2006)	All HIV+ women and an equivalent number of HIV− women who delivered by elective c-section.	Yes	Wound sepsis (not clearly defined)	3.8 *(52)*	5.8 *(52)*	0.65 (0.10–4.08)	-
**Fiore ** ***et al.,*** ** 2004 ** [**55**]	Prospective Cohort	14 references centres in Italy, Spain, Sweden, Poland and Ukraine (1992–2003)	All HIV+ women delivering by elective c-section were matched with the first HIV− woman delivering by elective c-section after the infected index case for age, ethnicity, parity, and whether admitted to the delivery unit in active labour.	Yes	Wound infection (no further details)	0.6 *(158)*	0 *(158)*	3.02 (0.12–74.67)	-
					Endometritis (no further details)	1.3 *(158)*	2.5 *(158)*	0.49 (0.09–2.73)	-
**Grubert ** ***et al*** **., 1999 ** [**18**]	Retrospective Cohort	One medical facility in Germany (1987–1999)	All HIV+ women delivering by c-section were matched to an HIV− woman on age, duration of gestation and indication for caesarean.	Yes	Endometritis (no further details)	4.8 *(62)*	0 *(62)*	7.35 (0.37–145.40)	-
**Louis ** ***et al.,*** ** 2007 ** [**26**]	Prospective Cohort	19 different academic medical centres in the USA (1999–2002)	All women of known HIV status having a c-section at a gestational age of >20 weeks and who delivered a singleton infant of at least 500g birth weight.	Yes	Maternal sepsis defined as the presence of positive blood cultures and cardiovascular decompensation	1.1 *(378)*	0.2 *(54,281)*	6.98 (2.55–19.15)	6.2 (2.3–17.0)^2^
					Wound infection defined as erythema of the incision accompanied by purulent drainage requiring wound care	2.1 *(378)*	1.3 *(54,281)*	1.67 (0.83–3.39)	1.6 (0.8–3.3)^2^
					Endometritis defined as persistently elevated postpartum body temperature with uterine tenderness in the absence of a non-uterine source	11.6 *(378)*	5.8 *(54,281)*	2.14 (1.56–2.93)	1.9 (1.3–2.6) 2
**Maiques ** ***et al.,*** ** 2010 ** [**14**]	Retrospective cohort	One referral hospital in Valencia, Spain (1997–2007)	All HIV+ women on ART and having a c-section; for every HIV+ woman the HIV− women who delivered by c-section before and after were selected as controls.	Yes	Wound infection or hematoma (no further details)	5.0 *(160)*	2.8 *(320)*	1.82 (0.69–4.81)	-
					Endometritis defined using clinical signs and a positive vaginal swab	0.6 *(160)*	0.6 *(320)*	1.00 (0.09–11.11)	-
**Maiques-Montesinos ** ***et al.*** **, 1999 ** [**15**]	Retrospective Cohort	One maternity hospital in Valencia, Spain (1987–1996)	All HIV+ women delivering by c-section were matched to HIV− women for indication for c-section, stage of labour, number of foetuses and date of delivery.	No	Sepsis (no further details)	4.4 *(45)*	0 *(90)*	10.40 (0.49–221.37)	-
					Wound infection or hematoma (no further details)	26.7 *(45)*	6.7 *(90)*	5.09 (1.76–14.69)	-
					Endometritis (no further details)	2.2 *(45)*	4.4 *(90)*	0.49 (0.05–4.50)	-
**Moodlier ** ***et al.*** **, 2007 ** [**49**]	Retrospective Cohort	One tertiary hospital in Durban, South Africa (2003–2004)	All women undergoing a c-section with known HIV status.	No^1^	Wound sepsis defined as the breakdown of the suture line as a result of a subcutaneous infectious process	5.4 *(186)*	8.0 *(175)*	0.65 (0.28–1.51)	-
					Endometritis defined as a sustained pyrexia (auxiliary temp greater than 38°C) post-delivery (excluding the first 24 hours)	5.9 *(186)*	1.1 *(175)*	5.44 (1.19–24.89)	-
**Panburana ** ***et al.,*** ** 2003 ** [**54**]	Prospective Cohort	One tertiary hospital in Bangkok, Thailand (1999–2001)	Do not provide specific details on how HIV+ and HIV− women were selected but did exclude women who had a preterm delivery.	Yes	Endometritis (no further details)	2.7 *(74)*	1.7 *(360)*	1.64 (0.32–8.28)	-
**Rodriguez ** ***et al.,*** ** 2001 ** [**27**]	Prospective Cohort	One facility in the USA (no further details provided) (1992–2000)	All HIV+ women delivering by c-section were matched to an HIV− woman on age, race, year of delivery and indication for c-section.	Yes	Sepsis (no further details)	1.2 *(86)*	0 *(86)*	3.04 (0.12–75.55)	-
					Wound infection defined as purulent drainage, induration or tenderness	7.0 *(86)*	4.7 *(86)*	1.54 (0.42–5.65)	-
					Postpartum endometritis defined as a temperature >38^o^C on two consecutive readings at an 8 hour interval, exclusive of the first 24 hours after delivery, with uterine tenderness, foul lochia, and no other apparent causes for fever	16.3 *(86)*	10.5 *(86)*	1.66 (0.68–4.08)	-
**Semprini ** ***et al.,*** ** 1995 ** [**20**]	Retrospective Cohort	Seven centres in Italy (1989–1993)	All HIV+ women delivering by c-section were matched to an HIV− woman on indication for c-section, active labour and whether they had ruptured membranes.	No	Sepsis (no further details)	0.6 *(156)*	0 *(156)*	3.02 (0.12–74.69)	-
					Wound infection (no further details)	8.3 *(156)*	1.9 *(156)*	4.64 (1.29–16.61)	-
					Febrile endometritis (no further details)	13.5 *(156)*	2.6 *(156)*	5.91 (1.98–17.65)	-
**Urbani ** ***et al.,*** ** 2001 ** [**50**]	Prospective Cohort	Two teaching hospitals in South Africa (1998)	307 women were enrolled irrespective of HIV status, and subsequently HIV status was ascertained. Women were excluded if they had diabetes mellitus.	No	Wound infection (no further details)	6.8 *(59)*	3.2 *(248)*	2.18 (0.63–7.51)	-
					Endometritis defined as fever of ≥38^o^C on 2 occasions at least 4 hours apart and more than 24 hours post-operatively, tachycardia of >100 beats per minute on 2 occasions at least 4 hours apart and more than 24 hours post-operatively, and tenderness of the cervix on movement	23.7 *(59)*	6.9 *(248)*	4.23 (1.95–9.19)	-
**Zvandasara ** ***et al.*** **, 2007 ** [**45**]	Prospective Cohort	One maternity hospital in Harare, Zimbabwe (2006)	All patients undergoing a c-section with known HIV status.	No	Wound infection was diagnosed in the presence of purulent discharge from the incision with induration and tenderness with or without fever	23.8 *(164)*	15.7 *(382)*	1.67 (1.06–2.63)	-
					Postpartum endometritis defined as temperature ≥38^o^C on 2 successive readings at an 8 hour interval (excluding the 24 hours after delivery) and uterine tenderness, slight vaginal bleeding or foul smelling odour and no other apparent causes of fever	25.6 *(164)*	20.9 *(382)*	1.30 (0.85–1.99)	-

1 Information was not supplied in the published paper so whether antiretroviral treatment should have been available was based on the study dates and study location; for one study it was not clear from the study dates and location whether ART would be available so the information was inferred from the literature.

a) Durban, South Africa in 2004: No ART treatment based on the UNAIDS data accessed on 20th December 2012 at http://www.unaids.org/en/regionscountries/countries/southafrica/.

2 Adjusted for number of previous caesarean section.

### Risk of Bias Within and Between Data Sets

The assessment of the risk of bias is summarised in Tables 3 and 4. Only 23 of the 66 data sets provided a definition for the obstetric complication: from eight of the 19 data sets (42%) reporting on hypertensive disorders to seven amongst the 25 data sets (28%) for intrauterine infections. The risk of bias in the ascertainment of obstetric complications cases was judged to be high for 29 of the 66 data sets; most of which relied on medical records to ascertain the nature of the complication.

**Table 3:** Risk of bias within studies which include vaginal deliveries.

Reference	Definition of obstetric complication		Ascertainment of obstetric complication		Completeness of data			Adjustment for confounders		Selection of comparison group
HAEMORRHAGE										
**Aboud ** ***et al.*** ** 2009 ** [**56**] ** with supplementary information from ** [**75**]	No definitions provided for antepartum haemorrhage or postpartum haemorrhage		The pregnancy was followed prospectively, although it was not clear who collected the outcome data		6.9% of HIV+ women were lost to follow up compared with 7.6% of HIV− women			None		Unclear on exact selection methods; however no HIV− women were selected from one of the study sites
	**High risk**		**Unclear risk**		**Low risk**			**High risk**		**High risk**
**Azria ** ***et al.*** **, 2010 ** [**16**]	Definition provided for postpartum haemorrhage		Hospital record review		Eight medical records of HIV+ women were missing data; no information for HIV− women			HIV+ and HIV− women were matched on key confounders		HIV+ and HIV− women selected from the same hospital
	**Low risk**		**High risk**		**Unclear risk**			**Low risk**		**Low risk**
**Braddick ** ***et al.,*** ** 1990 ** [**32**]	Definition provided for antepartum haemorrhage		Hospital record review		0.6% of women refused to participate			None		HIV− Women were selected based on close proximity to the follow up clinic, but this selection criteria was not applied to HIV+ women
	**Low risk**		**High risk**		**Low risk**			**High risk**		**High risk**
**Chamiso, 1996 ** [**36**]	No definitions provided for placenta praevia, postpartum haemorrhage or retained placenta		Recorded by a general practitioner blinded to woman's HIV status		22% of HIV+ women were lost to follow up			HIV+ and HIV− women were matched on key confounders		HIV+ and HIV− women selected from the same hospital
	**High risk**		**Low risk**		**High risk**			**Low risk**		**Low risk**
**Chanrachakul ** ***et al.,*** ** 2001 ** [**53**]	No definitions provided for postpartum haemorrhage or retained placenta		Hospital record review		No information provided			None		HIV+ and HIV− women were enrolled from the same hospital; however HIV+ women were managed using traditional labour management and HIV− women were managed using active labour management
	**High risk**		**High risk**		**Unclear risk**			**High risk**		**High risk**
**De Groot ** ***et al.,*** ** 2003 ** [**46**]	Definitions provided for antepartum haemorrhage and postpartum haemorrhage		Hospital record review		2% of medical files selected into study were missing HIV status			None		HIV+ and HIV− women selected from the same hospital
	**Low risk**		**High risk**		**Low risk**			**High risk**		**Low risk**
**Figueroa-Damian, 1999 ** [**28**]	No definition provided for postpartum haemorrhage		The pregnancy was followed prospectively, although it was not clear who collected the outcome data		No information provided			HIV+ and HIV− women were matched on key confounders		HIV+ and HIV− women selected from the same hospital
	High risk		Unclear risk		Unclear risk			Low risk		Low risk
**Haeri ** ***et al.,*** ** 2009 ** [**21**]	No definition provided for placental abruption		Hospital record review		No information provided			HIV+ and HIV− women were matched on key confounders		HIV+ and HIV− women selected from the same two tertiary care centres
	**High risk**		**High risk**		**Unclear risk**			**Low risk**		**Low risk**
**Kourtis ** ***et al.,*** ** 2006 ** [**22**]	ICD-9 codes were used to define antepartum haemorrhage		Hospital discharge data		No information provided			None^1^		HIV+ and HIV− women selected from the same hospitals
	**Low risk**		**High risk**		**Unclear risk**			**High risk**		**Low risk**
**Leroy ** ***et al*** **., 1998 ** [**37**]	No definitions provided for postpartum haemorrhage or retained placenta		Recorded by a midwife blinded to woman's HIV status		5.2% refusal rate; 4.7% lost to follow up			HIV+ and HIV− women were matched on key confounders		HIV+ and HIV− women selected from the same hospital
	**High risk**		**Low risk**		**Low risk**			**Low risk**		**Low risk**
**Lionel ** ***et al*** **., 2008 ** [**51**]	Placental abruption defined as grade III but no definitions for placenta praevia and PPH		Hospital record review		No information provided			None		HIV+ and HIV− women selected from the same hospital
	**Unclear risk**		**High risk**		**Unclear risk**			**High risk**		**Low risk**
**Louis ** ***et al.,*** ** 2006 ** [**23**]	No definition provided for postpartum haemorrhage		Hospital record review		No information provided			None		HIV+ and HIV− women selected from the same hospital
	**High risk**		**High risk**		**Unclear risk**			**High risk**		**Low risk**
**Minkoff ** ***et al.,*** ** 1990 ** [**24**]	No definitions provided for placenta praevia, placental abruption, peripartum haemorrhage or retained placenta		Method of ascertaining outcome not clear		10% of HIV+ women refused to participate			None		HIV+ and HIV− women selected from the same clinics
	**High risk**		**Unclear risk**		**Low risk**			**High risk**		**Low risk**
**Mmiro ** ***et al*** **., 1993 ** [**39**]	No definitions provided for antepartum haemorrhage or postpartum haemorrhage		Hospital record review		No information provided			None		HIV+ and HIV− women selected from the same hospital
	**High risk**		**High risk**		**Unclear risk**			**High risk**		**Low risk**
**Peret ** ***et al*** **., 1993 ** [**30**]	Definition provided for postpartum haemorrhage		The pregnancy was followed prospectively, although it was not clear who collected the outcome data		No information provided			HIV+ and HIV− women were matched on key confounders		HIV+ and HIV− women selected from the same hospital
	**Low risk**		**Unclear risk**		**Unclear risk**			**Low risk**		**Low risk**
**Van Eijk ** ***et al.*** **, 2007 ** [**33**]	No definition provided for peripartum haemorrhage		Method of ascertaining outcome not clear		1.5% of women had missing data and were excluded			None		HIV+ and HIV− women selected from the same hospital
	**High risk**		**Unclear risk**		**Low risk**			**High risk**		**Low risk**
HYPERTENSIVE DISEASES OF PREGNANCY										
**Aboud ** ***et al.*** ** 2009 ** [**56**] ** with supplementary information from ** [**75**]	No definition provided for hypertension		The pregnancy was followed prospectively, although it was not clear who collected the outcome data		6.9% of HIV+ women were lost to follow up compared with 7.6% of HIV− women			None		Unclear on exact selection methods; however no HIV− women were selected from one of the study sites
	**High risk**		**Unclear risk**		**Low risk**			**High risk**		**High risk**
**Bodkin ** ***et al.,*** ** 2005 ** [**47**]	No definitions provided for pregnancy-induced hypertension or eclampsia		Hospital record review		49.6% of women refused to be tested for HIV			HIV+ and HIV− women were only matched on whether their pregnancy was high-risk, medium-risk or low-risk		HIV+ and HIV− women selected from the same hospital
	**High risk**		**High risk**		**High risk**			**High risk**		**Low risk**
**Boer ** ***et al.,*** ** 2006 ** [**19**]	Definition provided for pre-eclampsia		Method of ascertaining outcome not clear		No information provided			HIV+ and HIV− women were matched on key confounders		HIV+ and HIV− women selected from the same medical centres
	**Low risk**		**Unclear risk**		**Unclear risk**			**Low risk**		**Low risk**
**Chamiso, 1996 ** [**36**]	Definition provided from pregnancy-induced hypertension		Recorded by a general practitioner blinded to woman's HIV status		22% of HIV+ women were lost to follow up			HIV+ and HIV− women were matched on key confounders		HIV+ and HIV− women selected from the same hospital
	**Low risk**		**Low risk**		**High risk**			**Low risk**		**Low risk**
**De Groot ** ***et al.*** **, 2003 ** [**46**]	Definitions provided for pre-eclampsia and eclampsia		Hospital record review		2% of medical files selected into study were missing HIV status			None		HIV+ and HIV− women selected from the same hospital
	**Low risk**		**High risk**		**Low risk**			**High risk**		**Low risk**
**Figueroa-Damian, 1999 ** [**28**]	No definition provided for acute hypertensive disorder of pregnancy		The pregnancy was followed prospectively, although it was not clear who collected the outcome data		No information provided			HIV+ and HIV− women were matched on key confounders		HIV+ and HIV− women selected from the same hospital
	**High risk**		**Unclear risk**		**Unclear risk**			**Low risk**		**Low risk**
**Frank ** ***et al.,*** ** 2004** [**48**]	Definitions provided for pregnancy-induced hypertension and pre-eclampsia but not for eclampsia		Hospital record review		27% of files reviewed did not have known HIV status			None		HIV+ and HIV− women selected from the same hospital and clinics
	**Unclear risk**		**High risk**		**High risk**			**High risk**		**Low risk**
**Haeri ** ***et al.,*** ** 2009 ** [**21**]	Definition provided for pre-eclampsia but not for gestational hypertension		Hospital record review		No information provided			HIV+ and HIV− women were matched on key confounders and adjusted analysis conducted		HIV+ and HIV− women selected from the same two tertiary care centres
	**Unclear risk**		**High risk**		**Unclear risk**			**Low risk**		**Low risk**
**Kourtis ** ***et al.,*** ** 2006 ** [**22**]	ICD-9 codes were used to define pre-eclampsia/ hypertensive disorders of pregnancy		Hospital discharge data		No information provided			None^1^		HIV+ and HIV− women selected from the same hospitals
	**Low risk**		**High risk**		**Unclear risk**			**High risk**		**Low risk**
**Lionel ** ***et al.*** **, 2008 ** [**51**]	No definitions provided for pre-eclampsia and eclampsia		Hospital record review		No information provided			None		HIV+ and HIV− women selected from the same hospital
	**High risk**		**High risk**		**Unclear risk**			**High risk**		**Low risk**
**Mattar ** ***et al*** **., 2004 ** [**31**]	Definition provided for pre-eclampsia		Hospital record review		No information provided			None		HIV+ and HIV− women selected from the same clinic
	**Low risk**		**High risk**		**Unclear risk**			**High risk**		**Low risk**
**Mmiro ** ***et al*** **., 1993 ** [**39**]	Definition provided for hypertension		Hospital record review		No information provided			None		HIV+ and HIV− women selected from the same hospital
	**Low risk**		**High risk**		**Unclear risk**			**High risk**		**Low risk**
**Olagbuji ** ***et al*** **., 2010 ** [**42**]	No definition provided for pregnancy-induced hypertension		Method of ascertaining outcome not clear		No information provided			State that HIV− women were matched to HIV+ women but do not state what the matching characteristics were		HIV+ and HIV− women selected from the same hospital
	**High risk**		**Unclear risk**		**Unclear risk**			**Unclear risk**		**Low risk**
**Roman-Poueriet ** ***et al*** **., 2009 ** [**29**]	No definition provided for pregnancy-induced hypertension		Hospital record review		No information provided			None		Women were recruited from a range of public, private clinics and hospitals and a specialist HIV clinic
	**High risk**		**High risk**		**Unclear risk**			**High risk**		**Unclear risk**
**Singh ** ***et al.,*** ** 2009 ** [**52**]	No definition provided for pre-eclamptic toxaemia		States that the “antenatal complications in both study groups were observed”		No information provided			None		HIV+ and HIV− women selected from the same hospital
	**High risk**		**Unclear risk**		**Unclear risk**			**High risk**		**Low risk**
**Suy ** ***et al.,*** ** 2006 ** [**13**]	Definition provided for pre-eclampsia		Using a database		No information provided			Adjusted analysis was conducted but it is not clear what factors were adjusted for, therefore only the crude estimate was extracted		HIV+ and HIV− women selected from the same hospital
	**Low risk**		**High risk**		**Unclear risk**			**High risk**		**Low risk**
**Waweru ** ***et al.*** **, 2009 ** [**34**]	No definition provided for pre-eclampsia		Method of ascertaining outcome not clear		No information provided			None		HIV+ and HIV− women selected from the same hospital
	**High risk**		**Unclear risk**		**Unclear risk**			**High risk**		**Low risk**
**Wimalasundera ** ***et al.*** **, 2002 ** [**17**]	Definition provided for pre-eclampsia		Method of ascertaining outcome not clear		No information provided			HIV+ and HIV− women were matched on key confounders		HIV+ and HIV− women selected from the same hospitals
	**Low risk**		**Unclear risk**		**Unclear risk**			**Low risk**		**Low risk**
DYSTOCIA										
**Chanrachakul ** ***et al.,*** ** 2001 ** [**53**]		Definition provided for prolonged labour		Hospital record review		No information provided	None		Although HIV+ and HIV− women were enrolled from the same hospital, HIV+ women were managed using traditional labour management and HIV− women were managed using active labour management	
		**Low risk**		**High risk**		**Unclear risk**	**High risk**		**High risk**	
**Leroy ** ***et al*** **., 1998 ** [**37**]		No definitions provided for dystocia or abnormal presentation		Recorded by a midwife blinded to woman's HIV status		5.2% refusal rate; 4.7% lost to follow up	HIV+ and HIV− women were matched on key confounders		HIV+ and HIV− women selected from the same hospital	
		**High risk**		**Low risk**		**Low risk**	**Low risk**		**Low risk**	
**Lionel ** ***et al.*** **, 2008 ** [**51**]		No definition provided for uterine rupture		Hospital record review		No information provided	None		HIV+ and HIV− women selected from the same hospital	
		**High risk**		**High risk**		**Unclear risk**	**High risk**		**Low risk**	
**Minkoff ** ***et al.,*** ** 1990 ** [**24**]		No definition provided for abnormal presentation		Method of ascertaining outcome not clear		10% of HIV+ women refused to participate	None		HIV+ and HIV− women selected from the same clinics	
		**High risk**		**Unclear risk**		**Low risk**	**High risk**		**Low risk**	
**Wandabwa ** ***et al.,*** ** 2008 ** [**40**]		Definition provided for uterine rupture		Cases of ruptured uterus were identified by clinical examination and at laparatomy		No information provided	Adjusted analysis conducted		HIV+ and HIV− women selected from the same hospital	
		**Low risk**		**Low risk**		**Unclear risk**	**Low risk**		**Low risk**	
INTRAUTERINE INFECTION										
**Aboud ** ***et al.*** ** 2009 ** [**56**] ** with supplementary information from ** [**75**]		No definition provided for puerperal sepsis		The pregnancy was followed prospectively, although it was not clear who collected the outcome data		6.9% of HIV+ women were lost to follow up compared with 7.6% of HIV− women	None		Unclear on exact selection methods; however no HIV− women were selected from one of the study sites	
		**High risk**		**Unclear risk**		**Low risk**	**High risk**		**High risk**	
**Chamiso, 1996 ** [**36**]		No definition provided for endometritis		Recorded by a general practitioner blinded to woman's HIV status		22% of HIV+ women were lost to follow up	HIV+ and HIV− women were matched on key confounders		HIV+ and HIV− women selected from the same hospital	
		**High risk**		**Low risk**		**High risk**	**Low risk**		**Low risk**	
**Chanrachakul ** ***et al.,*** ** 2001 ** [**53**]		No definition provided for puerperal infection		Hospital record review		No information provided	None		Although HIV+ and HIV− women were enrolled from the same hospital, HIV+ women were managed using traditional labour management and HIV− women were managed using active labour management	
		**High risk**		**High risk**		**Unclear risk**	**High risk**		**High risk**	
**Figueroa-Damian, 1999 ** [**28**]		No definition provided for endometritis		The pregnancy was followed prospectively, although it was not clear how the outcome data was collected		No information provided	HIV+ and HIV− women were matched on key confounders		HIV+ and HIV− women selected from the same hospital	
		**High risk**		**Unclear risk**		**Unclear risk**	**Low risk**		**Low risk**	
**Fiore ** ***et al.,*** ** 2004 ** [**55**]		No definition provided for endometritis		Women were evaluated for the development of obstetric complications		No information provided	HIV+ and HIV− women were matched on key confounders		HIV+ and HIV− women selected from the same delivering centres	
		**High risk**		**Low risk**		**Unclear risk**	**Low risk**		**Low risk**	
**Kourtis ** ***et al.,*** ** 2006 ** [**22**]		ICD-9 codes used to define major puerperal sepsis		Hospital discharge data		No information provided	None^1^		HIV+ and HIV− women selected from the same hospitals	
		**Low risk**		**High risk**		**Unclear risk**	**High risk**		**Low risk**	
**Lepage ** ***et al.*** ** 1991 ** [**38**]		No definition provided for endometritis		Hospital record review		21% refusal rate; 16% of HIV− women and 21% of HIV+ women were lost to follow up	HIV+ and HIV− women were matched on key confounders		HIV+ and HIV− women selected from the same hospital	
		**High risk**		**High risk**		**High risk**	**Low risk**		**Low risk**	
**Minkoff ** ***et al.,*** ** 1990 ** [**24**]		No definition provided for endometritis		Method of ascertaining outcome not clear		10% of HIV+ women refused to participate	None		HIV+ and HIV− women selected from the same clinics	
		**High risk**		**Unclear risk**		**Low risk**	**High risk**		**Low risk**	
**Okong ** ***et al.*** ** 2004 ** [**41**]		Definition provided for endometritis		Cases of endometritis were identified by midwives		8% of cases and controls refused to participate	None		HIV+ and HIV− women selected from the same hospital	
		**Low risk**		**Low risk**		**Low risk**	**High risk**		**Low risk**	
**Onah ** ***et al.*** **, 2007 ** [**43**]		No definition provided for puerperal sepsis		Hospital record review		19% of HIV− women had to be excluded because their medical records could not be located	None		HIV+ and HIV− women selected from the same hospital	
		**High risk**		**High risk**		**Low risk**	**High risk**		**Low risk**	
**Peret ** ***et al*** **., 1993 ** [**30**]		Definition provided for endometritis		The pregnancy was followed prospectively, although it was not clear who collected the outcome data		No information provided	HIV+ and HIV− women were matched on key confounders		HIV+ and HIV− women selected from the same hospital	
		**Low risk**		**Unclear risk**		**Unclear risk**	**Low risk**		**Low risk**	
**Temmerman ** ***et al.,*** ** 1994 ** [**35**]		Definition provided for endometritis		Identified by a research nurse		2.3% women refused to participate; missing data for 38% of HIV+ and 35% of HIV− women on endometritis	HIV+ and HIV− women were matched on key confounders		HIV+ and HIV− women selected from the same health centre	
		**Low risk**		**Low risk**		**High risk**	**Low risk**		**Low risk**	

1 Adjusted odds ratios stratified by two time periods were available but were not extracted.

**Table 4:** Risk of bias within caesarean section studies.

Reference	Definition of obstetric complication		Ascertainment of obstetric complication		Completeness of data			Adjustment for confounders		Selection of comparison group
HAEMORRHAGE										
**Chama and Morrupa, 2008 ** [**44**]	Definition provided for intra-operative haemorrhage		No information provided		No information provided			None		HIV+ and HIV− women selected from the same hospital
	**Low risk**		**Unclear risk**		**Unclear risk**			**High risk**		**Low risk**
HYPERTENSIVE DISEASES OF PREGNANCY										
**Chama and Morrupa, 2008 ** [**44**]	No definition provided for pregnancy-induced hypertension		No information provided		No information provided			None		HIV+ and HIV− women selected from the same hospital
	**High risk**		**Unclear risk**		**Unclear risk**			**High risk**		**Low risk**
INTRAUTERINE INFECTION										
**Cavasin ** ***et al.,*** ** 2009 ** [**25**]		Definition provided for endometritis, but not for septic pelvic thrombosis		Hospital record review		No information provided	None		HIV+ and HIV− women selected from the same two health centres	
		**Unclear risk**		**High risk**		**Unclear risk**	**High risk**		**Low risk**	
**Chama and Morrupa, 2008 ** [**44**]		No definition provided for wound sepsis		No information provided		No information provided	None		HIV+ and HIV− women selected from the same hospital	
		**High risk**		**Unclear risk**		**Unclear risk**	**High risk**		**Low risk**	
**Fiore ** ***et al.,*** ** 2004 ** [**55**]		No definitions provided for wound infection or endometritis		Women were evaluated for the development of obstetric complications		No information provided	HIV+ and HIV− women were matched on key confounders		HIV+ and HIV− women selected from the same delivering centres	
		**High risk**		**Low risk**		**Unclear risk**	**Low risk**		**Low risk**	
**Grubert ** ***et al*** **., 1999 ** [**18**]		No definition provided for endometritis		Method of ascertaining outcome not clear		No information provided	HIV+ and HIV− women were matched on key confounders		HIV+ and HIV− women selected from the same medical facility	
		**High risk**		**Unclear risk**		**Unclear risk**	**Low risk**		**Low risk**	
**Louis ** ***et al.,*** ** 2007 ** [**26**]		Definitions provided maternal sepsis, wound infection and endometritis		Method of ascertaining outcome not clear		No information provided	Adjusted analysis conducted		HIV+ and HIV− women selected from the same medical centres	
		**Low risk**		**Unclear risk**		**Unclear risk**	**Low risk**		**Low risk**	
**Maiques ** ***et al.,*** ** 2010 ** [**14**]		Definition provided for endometritis, but not for wound infection		Method of ascertaining outcome not clear		No information provided	None		HIV+ and HIV− women selected from the same hospital	
		**Unclear risk**		**Unclear risk**		**Unclear risk**	**High risk**		**Low risk**	
**Maiques-Montesinos ** ***et al.*** **, 1999 ** [**15**]		No definitions provided for sepsis, wound infection/haematoma or endometritis		Hospital record review		No information provided	HIV+ and HIV− women were matched on key confounders		HIV+ and HIV− women selected from the same hospital	
		**High risk**		**High risk**		**Unclear risk**	**Low risk**		**Low risk**	
**Moodlier ** ***et al.*** **, 2007 ** [**49**]		Definitions provided wound sepsis and endometritis		Hospital record review		Only 1% of charts were missing, but state that about half of the women refused HIV testing	None		HIV+ and HIV− women selected from the same hospital	
		**Low risk**		**High risk**		**High risk**	**High risk**		**Low risk**	
**Panburana ** ***et al.,*** ** 2003 ** [**54**]		No definition provided for endometritis		Simply states that all complications were “recorded in both study and control groups”		No information provided	None		HIV+ and HIV− women selected from the same hospital	
		**High risk**		**Unclear risk**		**Unclear risk**	**High risk**		**Low risk**	
**Rodriguez ** ***et al.,*** ** 2001 ** [**27**]		Definition provided for endometritis and wound infection, but not for sepsis		Hospital record review		11% of HIV+ women did not have records available for review	HIV+ and HIV− women were matched on key confounders		HIV+ and HIV− women selected from the same hospital	
		**Unclear risk**		**High risk**		**Low risk**	**Low risk**		**Low risk**	
**Semprini ** ***et al.,*** ** 1995 ** [**20**]		No definitions provided for sepsis, wound infection or febrile endometritis		Method of ascertaining outcome not clear		No information provided	HIV+ and HIV− women were matched on key confounders		HIV+ and HIV− women selected from the same centres	
		**High risk**		**Unclear risk**		**Unclear risk**	**Low risk**		**Low risk**	
**Urbani ** ***et al.,*** ** 2001 ** [**50**]		Definition provided for endometritis but not for wound infection		Identified by a researcher		95% of women undergoing c-section were recruited	None		HIV+ and HIV− women selected from the same hospitals	
		**Unclear risk**		**Low risk**		**Low risk**	**High risk**		**Low risk**	
**Zvandasara ** ***et al.*** **, 2007 ** [**45**]		Definition provided for wound sepsis		Identified by a researcher		No patients were excluded	None		HIV+ and HIV− women selected from the same hospital	
		**Low risk**		**Low risk**		**Low risk**	**High risk**		**Low risk**	

Very few studies had sufficient information on the completeness of the data to enable the risk of bias to be assessed and only 17 of 66 data sets were classified as at low risk of bias. In particular, studies relying on medical records tended not to report how many records had to be excluded due to missing information (e.g. HIV status).

Overall, 25 of 66 data sets either adjusted for confounders in their analysis or matched the HIV-infected and uninfected women with respect to some key confounders. The majority of the data sets (58 of 66) were judged to be at low risk of bias in the selection of the comparison group of HIV-uninfected women.

There was no evidence of publication bias for any of the outcomes included in the analysis with the exception of pre-eclampsia (p = 0.01) (Supplementary material, Figure S1).

### Effect of HIV on obstetric haemorrhage

**Figure 2:**
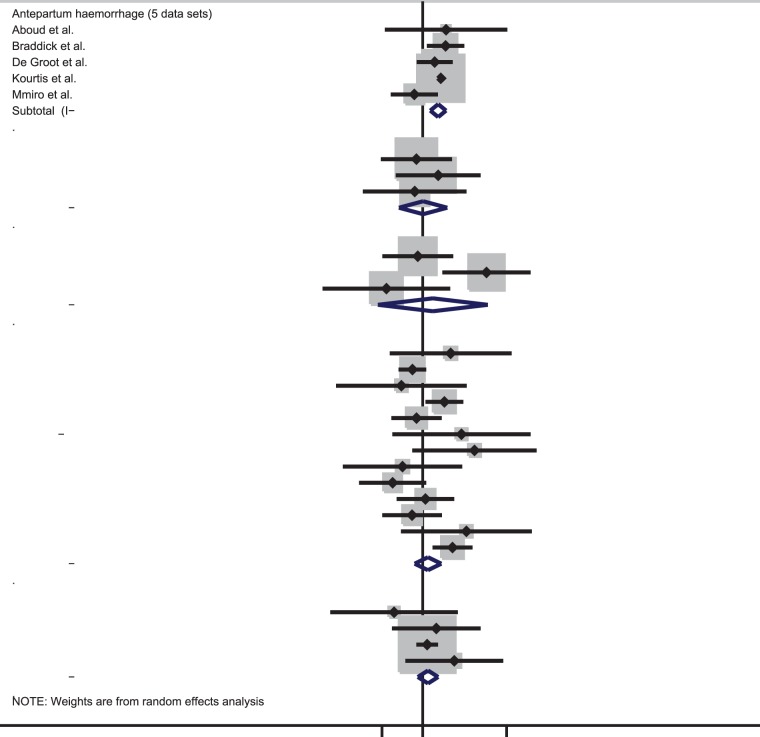
Forest plot showing the strength of association between HIV and obstetric haemorrhage in studies with vaginal deliveries only or included both vaginal deliveries and c-section deliveries.

The prevalence of antepartum haemorrhage was higher in HIV-infected than uninfected women in four out of five data sets (Table 1). Meta-analysis indicated that HIV-infected women have double the odds of antepartum haemorrhage [summary odds ratio (OR): 2.06, 95% confidence interval (CI): 1.42–2.97] (Figure 2). There was no evidence for between-study heterogeneity (I^2^: 27.5%, p-value = 0.24). Based on three data sets, there was no evidence for an association between HIV and either placenta praevia (summary OR: 1.02, 95% CI: 0.33–3.14, I^2^: 0%, p-value = 0.70) or placental abruption (summary OR: 1.61, 95% CI: 0.12–20.79, I^2^: 76.1%, p-value = 0.02) (Figure 2).

Thirteen data sets compared the prevalence of postpartum haemorrhage in HIV-infected and uninfected women with ORs ranging from 0.25 to 11.18. The meta-analysis suggests there is no evidence that HIV increases the odds of postpartum haemorrhage (summary OR: 1.28, 95% CI: 0.69–2.38, I^2^: 53.4%, p = 0.01). Similarly, there was no evidence for increased odds of retained placenta with HIV infection (summary OR: 1.28, 95% CI: 0.80–2.06, I^2^: 0%, p = 0.50).

One study looked at the association between HIV and postpartum haemorrhage amongst women undergoing a caesarean section (Table 2). There was no evidence of an association between HIV and postpartum haemorrhage (OR: 0.44, 95%CI: 0.19–1.04).

### Effect of HIV on hypertensive disorders of pregnancy

**Figure 3:**
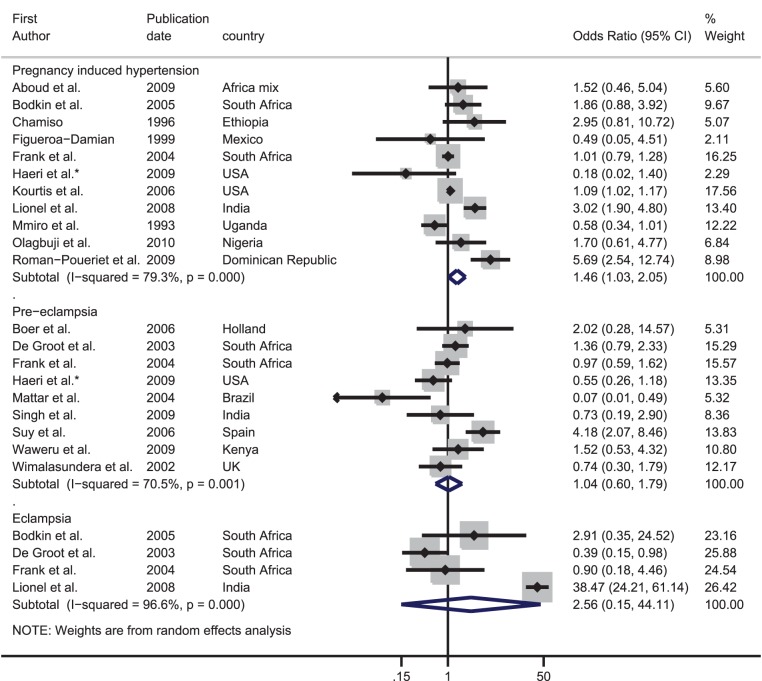
Forest plot showing the strength of association between HIV and hypertensive diseases of pregnancy. ^*^Adjusted odds ratio.

Out of the 11 data sets with data on pregnancy-induced hypertension, eight found that HIV-infected women were at increased risk of pregnancy-induced hypertension (Table 1). The meta-analysis showed some evidence for increased odds of pregnancy-induced hypertension with HIV infection (summary OR: 1.46, 95% CI: 1.03–2.05). However, there was strong evidence for between-study heterogeneity (I^2^: 79.3%, p-value<0.001) (Figure 3).

Nine data sets examined the association between HIV and pre-eclampsia; four of these found a higher prevalence in HIV-infected women than uninfected women. There was no evidence that HIV infection was associated with pre-eclampsia (summary OR: 1.04, 95% CI: 0.60–1.79, I^2^: 70.5%, p-value = 0.001).

The ORs from the four data sets comparing the prevalence of eclampsia in HIV-infected and uninfected women varied from 0.39 to 38.47. The meta-analysis produced a summary OR of 2.56, however the confidence intervals were very wide (95% CI: 0.15–44.11) and there was strong evidence for between-study heterogeneity (I^2^: 96.6%, p-value<0.001).

There was one data set from Nigeria which was restricted to caesarean sections. There was no evidence of an association between HIV and postpartum pregnancy-induced hypertension (OR: 0.33, 95%CI 0.01–8.21).

### Effect of HIV on dystocia

**Figure 4:**
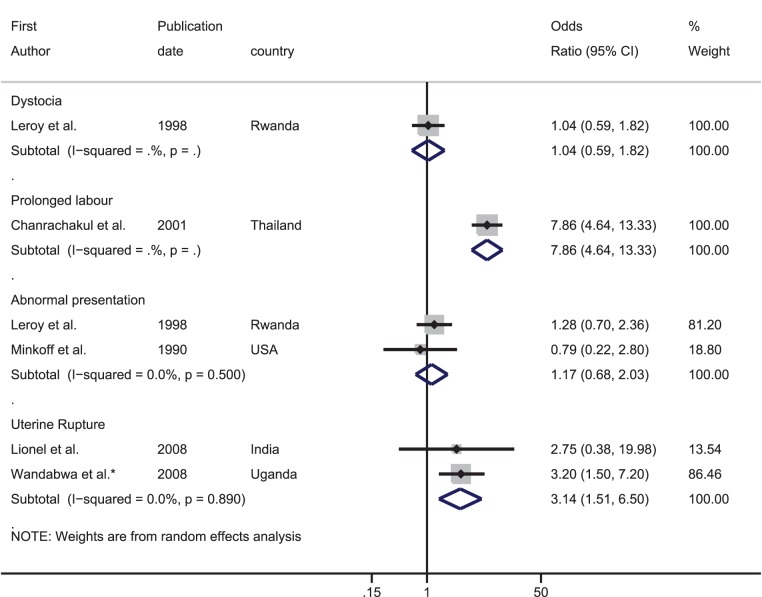
Forest plot showing the strength of association between HIV and dystocia. ^*^Adjusted odds ratio.

There were only six data sets where the outcome could be broadly categorised as dystocia (Table 1, Figure 4). One data set from Rwanda found no association between HIV and dystocia (OR: 1.04, 95% CI 0.59–1.82), whilst a study from Thailand indicated that HIV-infected women have nearly eight times the odds of prolonged labour compared with uninfected women (OR: 7.86, 95% CI: 4.64–13.33). Two data sets reported on abnormal presentation, and there was no evidence for an association between HIV and abnormal presentation in the meta-analysis (summary OR: 1.17, 95% CI: 0.68–2.03, I^2^: 0%, p = 0.50). Conversely, both data sets which compared the prevalence of uterine rupture showed an increased risk in HIV-infected women, giving a summary OR of 3.14 (95% CI: 1.51–6.50, I^2^:0%, p = 0.89).

### Effect of HIV on intrauterine infections

**Figure 5:**
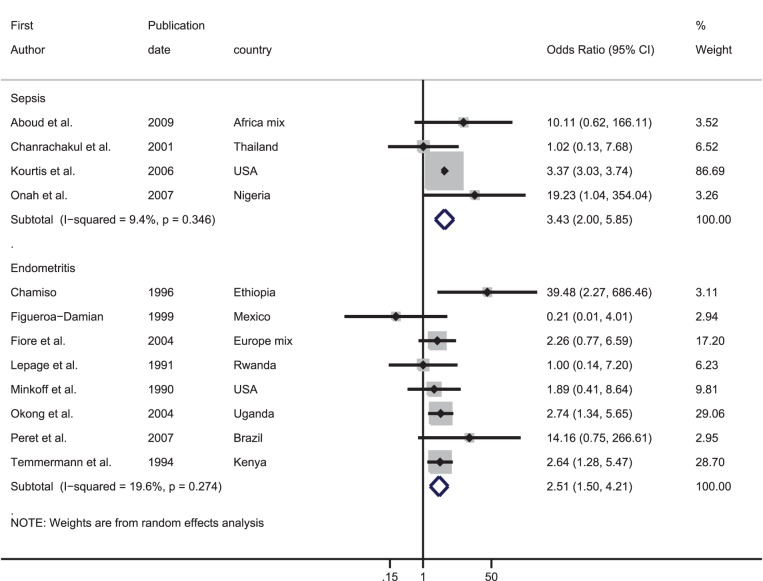
Forest plot showing the strength of association between HIV and intrauterine infection.

Figure 5 shows the association between HIV and intrauterine infections. Meta-analysis based on four data sets indicated that HIV-infected women have over three times the odds of having puerperal sepsis compared with uninfected women (summary OR 3.43, 95% CI: 2.00–5.85, I^2^: 9.4%, p-value = 0.35). There was also evidence from eight data sets that HIV-infected women had over 2.5 times the risk of endometritis compared with uninfected women (summary OR 2.51, 95% CI: 1.50–4.21, I^2^: 19.6%, p-value = 0.27).

**Figure 6:**
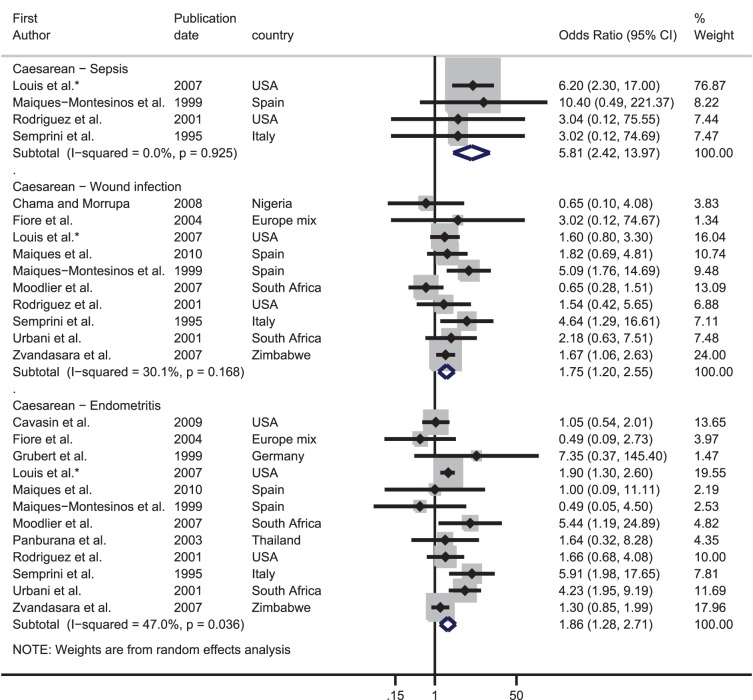
Forest plot showing the strength of association between HIV and intrauterine infection in studies which only looked at caesarean deliveries. ^*^Adjusted odds ratio.

The results of the meta-analyses for women who had a caesarean section are presented in Figure 6. The pooled OR from four data sets indicated that HIV-infected women had nearly six times higher odds of suffering from puerperal sepsis compared with their uninfected counterparts (summary OR 5.81, 95% CI: 2.42–13.97, I^2^: 0%, p-value = 0.93). Amongst the ten data sets which contained information on wound infection, the pooled OR was 1.75 (95% CI: 1.20–2.55) although there was weak evidence for between-study heterogeneity (I^2^: 30.1%, p = 0.17). Finally, there were 12 studies which looked at endometritis in HIV-infected and uninfected women; nine found a higher occurrence in HIV-infected women. The meta-analysis showed that HIV-infected women had over double the odds of endometritis than uninfected women (OR: 1.86, 95% CI: 1.28–2.71). There was good evidence for between-study heterogeneity (I^2^: 47.0%, p = 0.04).

### Caesarean section

**Figure 7:**
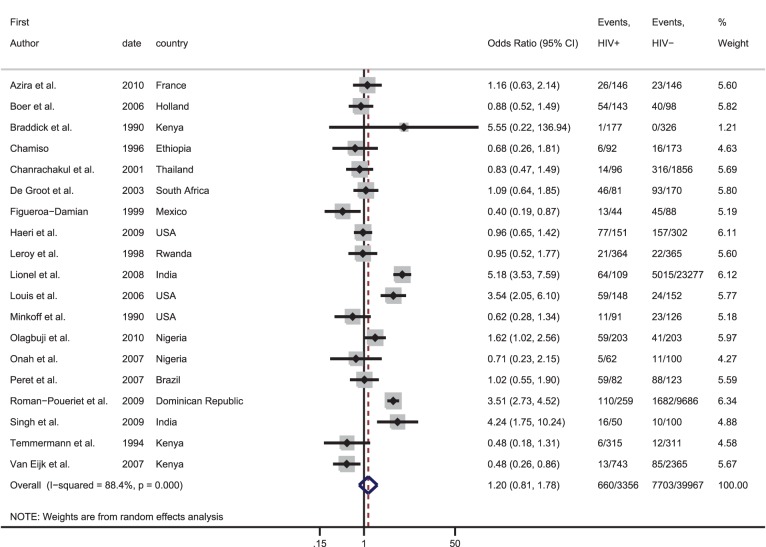
Forest plot showing the strength of association between HIV and caesarean section in studies included in this systematic review.

Of the studies which included vaginal and caesarean section deliveries, 13 did not provide information on the proportion of HIV-infected and uninfected women who had a caesarean (one stated that there was no difference in the mode of delivery in HIV-infected and uninfected women, one was restricted to only vaginal deliveries, three only followed women during pregnancy and eight did not provide any information on the proportion of infected and uninfected women having caesareans). Figure 7 shows the relative odds of having a caesarean for HIV-infected compared with uninfected women across the 19 studies which provided data. The ORs varied from 0.40 to 5.55 and there was no evidence that HIV-infected women were more likely to have a caesarean compared with uninfected women [pooled OR: 1.20, 95% CI: 0.81–1.78]. However, there was strong evidence for between-study heterogeneity (I^2^: 88.4%, p<0.001).

## Discussion

Our systematic review suggests that HIV increases the risk of intrauterine infections during pregnancy, delivery or the postpartum. Studies including vaginal and caesarean deliveries indicated that HIV-infected women had over three times the risk of a puerperal sepsis compared with uninfected women; this figure increased to nearly six amongst studies only including women who delivered by caesarean. The evidence for an association between HIV and other direct obstetric complications was inconsistent. Whilst HIV was associated with an increased risk of antepartum haemorrhage, there was no evidence of an increased risk of placenta praevia, placental abruption, postpartum haemorrhage or retained placenta. Similarly, HIV did appear to increase the risk of pregnancy-induced hypertension, but not of pre-eclampsia and eclampsia. Finally, we found an association between HIV and both uterine rupture and prolonged labour, but not between HIV and other complications of dystocia.

The higher risk of intrauterine infections in HIV-infected women is biologically plausible, as the immune suppression associated with HIV increases susceptibility to infection [57]. Caesarean sections may increase the risk of postpartum infection, but caesarean sections were equally common in HIV-infected and uninfected women, and the excess risk of intrauterine infections in HIV-infected women persisted among caesarean only deliveries. Whether the excess risk of endometritis and puerperal sepsis in the intra- and postpartum period is directly attributable to the pregnancy or indirectly related to HIV or AIDS-associated infections is uncertain. Intrauterine infections were mostly ascertained from hospital records, definitions were lacking, it was not always clear whether the infection was diagnosed during pregnancy or the postpartum and microbiological examination was not done. The signs and symptoms suggestive of endometritis and puerperal sepsis in intra- or postpartum women may have been a direct consequence of the increased prevalence of sexually transmitted infections associated with HIV [58], [59]. In the 2008–2010 confidential enquiries into maternal deaths in South Africa, only 6% of maternal deaths in HIV-infected women were attributed to pregnancy-related sepsis, while 62% of deaths were attributed to non-pregnancy-related infections [60]. Without clear definitions misclassification of non-pregnancy-related infections as pregnancy-related, or vice versa, cannot be excluded.

We did not find a consistent association between HIV and the risk of either haemorrhage, dystocia or hypertensive diseases of pregnancy. HIV-related thrombocytopenia affects around 10% of HIV-infected individuals and 30% of individuals with AIDS, [61]–[63] but it rarely leads to severe bleeding [61], [63]. The association between HIV and uterine rupture, concomitant with no association between HIV and other categories of dystocia, may suggest that delayed care seeking in HIV-infected women plays a role. However, this finding was based on two studies only, and caution is required in its interpretation. The observed associations between HIV and broadly defined categories of complications such as antepartum haemorrhage or hypertensive diseases – whereas no association was found between HIV and more narrowly defined clinical diagnoses such as placenta praevia, placental abruption, pre-eclampsia or eclampsia – suggests that measurement errors may have occurred. Unfortunately, few studies provided information on the number of women with more than one diagnosis, and we were not able to pool findings within the broad obstetric categories.

The lack of an association between HIV and caesarean section is surprising. Caesarean sections have been recommended to prevent the mother-to-child transmission (PMTCT) of HIV in many regions, [64], [65] and the most common indication for caesarean section in HIV-infected women in the studies reviewed was PMTCT of HIV (data not shown). It is possible that clinicians, particularly in low income countries, weigh the health risks associated with caesarean sections against those of PMTCT, and are perhaps more cautious about performing caesarean sections in HIV-infected women. Although recent guidelines recommend that women with very low viral loads who are on ART do not need a caesarean for PMTCT of HIV [66] we would expect a higher rate of caesareans amongst HIV-infected women in high-income countries given the time period in which the studies were conducted. Stratifying the meta-analysis by high and low income countries did not alter the findings (data not shown). We did not systematically review the literature to assess the association between HIV and caesarean sections, however, and some studies may have been missed.

This review was comprehensive covering a long time period with no restriction on language, world region or type of study. The studies found were predominantly conducted in tertiary health facilities, however, resulting in the enrolment of a higher risk group of pregnant women. While this will lead to an overestimation in the frequency of obstetric complications, it is unlikely this will have affected the relative odds comparing HIV-infected and uninfected women. The main limitation of this review is the poor quality of included studies, none of which were classified as at low risk of bias across all the quality components. Notably, only 25 data sets controlled for key confounders, either through matching HIV-infected and uninfected women or through adjustment in the analysis. Furthermore, very few studies provided information on how the obstetric complications were defined or ascertained. Whether the risks and stigma associated with HIV may result in health professionals reporting complications differentially in HIV-infected and uninfected women is not known, but information bias certainly needs considering. Unfortunately, due to the limited number of studies included in this review, it was not possible to tease out the effect of ART by restricting analyses to studies conducted when ART was available.

HIV-infected women are thought to be eight times more likely to die in pregnancy or the postpartum than HIV-uninfected women, [67], [68] and the excess mortality attributable to HIV among HIV-infected women is about 994 per 100,000 pregnant women [67]. While the increased risk of puerperal sepsis and endometritis in HIV-infected women contributes to this, direct obstetric causes only explain a tiny fraction of the excess mortality. Studies on the causes of death in pregnant or postpartum women by HIV status are scarce, except for the South African confidential enquiries, the most recent of which cover 2,756 and 1,149 maternal deaths in HIV-infected and uninfected women respectively [60]. The 2008–2010 confidential enquiries suggest that most deaths in HIV-infected pregnant and postpartum women are due to non-pregnancy-related infections, including pneumonia, tuberculosis and meningitis [60]. Although anaemia is thought to be exacerbated by HIV, [7] the confidential enquiries find a similar proportion of maternal deaths attributable to severe anaemia in HIV-infected and uninfected women (8% and 10% respectively) [60].

It is essential to ensure that both HIV-infected and uninfected pregnant woman have ready access to high quality antenatal and delivery services to correctly diagnose and manage direct obstetric complications when they occur. HIV-infected pregnant women will also benefit from prophylactic antibiotics during labour to reduce their risk of intrauterine infections [69]. However, given that most of the excess mortality associated with HIV in pregnancy is directly related to HIV rather than to a higher risk of obstetric complications, the greatest impact on pregnancy-related mortality will come from ensuring that HIV-infected pregnant women have adequate access to ART [70]. The World Health Organization recommends the provision of lifelong ART treatment for all HIV-infected pregnant women with a CD4 count below 350 cells/mm^3^, but many countries are still transitioning to these guidelines [71], [72]. Scaling up Option B+, where all pregnant mothers start ART regardless of their CD4 cell count and then continue taking it for life, has been proposed as an additional strategy to benefit maternal health; however, any benefit must be carefully measured against the potential pitfalls which include the high financial costs of such a programme and possible poor adherence to ART amongst women who perceive themselves to be healthy [73].

## Supporting Information

Figure S1 **Funnel plot illustrating potential publication bias for data sets which look at the association between HIV and pre-eclampsia.**
(EPS)

File S1 **Search Strategy.**
(DOCX)
