# Supplementary material for: HIV and the Risk of Direct Obstetric Complications: A Systematic Review and Meta-Analysis
Source: PLoS One. 2013 Oct 4;8(10):e74848. doi: 10.1371/journal.pone.0074848 (PMC3790789; doi:10.1371/journal.pone.0074848)
Supplement: File S1 — Search Strategy. (DOCX) [file pone.0074848.s002.docx]

**Supplementary Files – File S1**

**PUBMEB SEARCH STRATEGY**

**Search 1: HIV terms**

HIV OR “human immunodeficiency virus” OR AIDS OR “acquired immunodeficiency syndrome” OR HIV/AIDS OR HIV[MeSH] OR “HIV Infections”[MeSH] or "acquired immunodeficiency syndrome"[MeSH]

**Search 2: Maternal/pregnancy terms**

matern* OR pregnan* OR childbirth OR intrapartum OR intra-partum OR postpartum OR post-partum OR puerperal OR puerperium OR parturition OR “expectant mother” OR “expectant mothers” OR “maternal health services”[MeSH] OR “delivery, obstetric”[MeSH] OR parturition[MeSH] OR pregnancy [MeSH] OR “Delivery, Obstetric”[MeSH] OR “postpartum period”[MeSH]

**Search 3: Objective 1 specific terms (mortality)**

mortalit* OR fatalit* OR “fatal outcome” OR death OR deaths OR death[MeSH] OR mortality[MeSH]

**Search 4: Objective 2 specific terms (obstetric complications)**

morbidit* OR “pregnancy complication” OR “complication of pregnancy” OR “obstetric complication” OR “obstetric labor complication” OR “obstetric labour complication” OR “adverse pregnancy outcome” OR ((postpartum OR post-partum) AND (haemorrhage OR hemorrhage)) OR ((obstetric) AND (haemorrhage OR hemorrhage)) OR hemorrhage OR “vaginal bleeding” OR ((antepartum OR ante-partum) AND (haemorrhage OR hemorrhage)) OR dystocia OR ((obstructed OR prolonged) AND (labour OR labor)) OR “retained placenta” OR “pregnancy induced hypertension” OR hellp OR eclampsia OR preeclampsia OR “pre-eclampsia” OR “gestational diabetes” OR “abruptio placent*” OR “placental abruption” OR “placenta previa” OR "placenta praevia" OR “ruptured uterus” OR sepsis OR septic OR septicemia OR septicemic OR endometritis OR “puerperal infection” OR “near miss” OR “near-miss” OR “caesarean section” OR c-section OR “caesarian section” OR “cesarean section” OR anaemia OR anemia OR “iron deficien*” OR “obstetric labor complications”[MeSH] OR “pregnancy complications”[MeSH] OR hemorrhage[MeSH] OR “postpartum haemorrhage”[MeSH] OR “uterine inversion”[MeSH] OR “uterine hemorrhage”[MeSH] OR dystocia[MeSH] OR "placenta, retained"[MeSH] OR "hypertension, pregnancy-induced"[MeSH] OR "hellp syndrome"[MeSH Terms] OR eclampsia[MeSH] OR pre-eclampsia[MeSH] OR "diabetes, gestational"[MeSH] OR "abruptio placentae"[MeSH] OR "placenta previa"[MeSH] OR "uterine rupture"[MeSH] OR sepsis[MeSH] OR "cesarean section"[MeSH Terms] OR "anemia"[MeSH Terms]

**Search 5: Objective 3 specific terms (HIV disease progression)**

“CD4 lymphocyte count” OR “CD4 count” OR (HIV AND “disease progression”) OR “HIV severity” OR “aids defining” OR “AIDS-related opportunistic Infections” OR “kaposi's sarcoma” OR lymphoma OR “wasting syndrome” OR cachexia OR “pneumocystis carinii” OR tuberculosis OR tb OR “symptomatic HIV” OR “opportunistic infection” OR “opportunistic infections” OR “CD4 lymphocyte count”[MeSH] OR “AIDS-related opportunistic Infections”[MeSH] OR "lymphoma"[MeSH] OR "cachexia"[MeSH] OR "tuberculosis"[MeSH]

**Search** 6: Objective 4 specific terms (HIV incidence)

seroconversion OR incidence OR "incidence"[MeSH] OR "HIV infections/transmission"[MeSH]

**FINAL SEARCH: (#1 AND #2 AND (#3 OR #4 OR #5 OR #6))**

**EMBASE SEARCH STRATEGY**

**Search 1: HIV terms**

HIV OR human immunodeficiency virus OR HIV infections OR AIDS OR acquired immunodeficiency syndrome OR HIV/AIDs OR exp human immunodeficiency virus/ OR exp human immunodeficiency virus infection/ OR exp acquired immune deficiency syndrome/

**Search 2: Maternal/pregnancy terms**

matern* OR mother* OR pregnan*OR childbirth OR intrapartum OR intra-partum OR postpartum OR post-partum OR puerperal OR puerperium OR parturition OR expectant mother* OR exp expectant mother/ OR exp birth/ OR exp childbirth/ OR exp pregnancy/ OR exp delivery/

**Search 3: Objective 1 specific terms (mortality)**

mortalit*OR maternal mortality OR fatalit* OR fatal outcome OR death* OR exp mortality/ OR exp maternal mortality/ OR exp fatality/ OR exp death/

**Search 4: Objective 2 specific terms (obstetric complications)**

morbidit* OR pregnancy complication OR complication of pregnancy OR obstetric complication OR obstetric labor complication OR obstetric labour complication OR adverse pregnancy outcome OR ((postpartum OR post-partum) AND (haemorrhage OR hemorrhage)) OR ((obstetric) AND (haemorrhage OR hemorrhage)) OR hemorrhage OR vaginal bleeding OR ((antepartum OR ante-partum) AND (haemorrhage OR hemorrhage)) OR dystocia OR ((obstructed OR prolonged) AND (labour OR labor)) OR retained placenta OR pregnancy induced hypertension OR hellp OR eclampsia OR preeclampsia OR pre-eclampsia OR gestational diabetes OR abruptio placent* OR placental abruption OR placenta previa OR placenta praevia OR ruptured uterus OR sepsis OR septic OR septicemia OR septicemic OR endometritis OR puerperal infection OR near miss OR near-miss OR caesarean section OR c-section OR caesarian section OR cesarean section OR anaemia OR anemia OR iron deficien* OR exp morbidity/ OR exp maternal morbidity/ OR exp pregnancy complication/ OR exp labor complication/ OR exp postpartum hemorrhage/ OR exp bleeding/ OR antepartum hemorrhage/ OR exp obstetric hemorrhage/ OR exp dystocia/ OR exp retained placenta/ OR exp maternal hypertension/ OR exp HELLP syndrome/ OR exp "eclampsia and preeclampsia"/ OR exp pregnancy diabetes mellitus/ OR exp placenta previa/ OR exp uterus rupture/ OR exp sepsis/ OR exp septic shock/ OR exp septicemia/ OR exp endometritis/ OR exp puerperal infection/ OR exp cesarean section/ OR exp anemia/ OR exp iron deficiency anemia/

**Search 5: Objective 3 specific terms (HIV disease progression)**

CD4 lymphocyte count OR CD4 count OR HIV disease progression OR HIV severity OR aids defining OR AIDS-related opportunistic Infections OR kaposi's sarcoma OR lymphoma OR wasting syndrome OR cachexia OR pneumocystis carinii OR tuberculosis OR tb OR symptomatic HIV OR opportunistic infection* OR exp CD4 lymphocyte count/ OR exp disease course/ OR exp AIDS related complex/ OR exp kaposi sarcoma/ OR exp lymphoma/ OR exp wasting syndrome/ OR exp cachexia/ OR exp pneumocystis carinii/ OR exp tuberculosis/ OR exp opportunistic infection/

**Search** 6: Objective 4 specific terms (HIV incidence)

seroconversion OR incidence OR exp seroconversion/ OR exp incidence/ OR exp disease transmission/

**FINAL SEARCH: (#1 AND #2 AND (#3 OR #4 OR #5 OR #6))**

**POPLINE SEARCH STRATEGY**

**Search 1: HIV terms**

(HIV/“human immunodeficiency virus” / AIDS / “acquired immunodeficiency syndrome” / “HIV Infections”)

**Search 2: Maternal/pregnancy terms**

(matern* / pregnan* / childbirth / intrapartum / intra-partum / postpartum / post-partum / puerperal / puerperium / parturition / “expectant mother” / “expectant mothers”)

**Search 3: Objective 1 specific terms (mortality)**

(mortalit* / fatalit* / death*)

**Search 4: Objective 2 specific terms (obstetric complications)**

(morbidit* / “pregnancy complication” / “obstetric complication” / “obstetric labor complication” / “obstetric labour complication” / “adverse pregnancy outcome” / “postpartum haemorrhage” / “postpartum hemorrhage” / “obstetric haemorrhage” / “obstetric hemorrhage” / hemorrhage / “vaginal bleeding” / “antepartum haemorrhage” / “antepartum hemorrhage” / dystocia / “obstructed labour” / “obstructed labor” / “prolonged labour” / “prolonged labor” / “retained placenta” / “pregnancy induced hypertension” / hellp / eclampsia / preeclampsia / “pre-eclampsia” / “gestational diabetes” / “abruptio placent*” / “placental abruption” / “placenta previa” / "placenta praevia" / “ruptured uterus” / sepsis / septic / septicemia / septicemic / endometritis / “puerperal infection” / “near miss” / “near-miss” / “caesarean section” / c-section / “caesarian section” / “cesarean section” / anaemia / anemia)

**Search 5: Objective 3 specific terms (HIV disease progression)**

(“CD4 lymphocyte count” / “CD4 count” ***/*** “HIV disease progression” / “HIV severity” / “aids defining” **/ *“***AIDS-related opportunistic Infections” / “kaposi's sarcoma” / lymphoma / “wasting syndrome” / cachexia / ***“***pneumocystis carinii” / tuberculosis / tb / “symptomatic HIV” / “opportunistic infection*”)

***Search*** 6: Objective 4 specific terms (HIV incidence)

(seroconversion / incidence)

**FINAL SEARCH (advanced search in title/keywords and abstract)**

(HIV/“human immunodeficiency virus” / AIDS / “acquired immunodeficiency syndrome” / “HIV Infections”) **&** (matern* / pregnan* / childbirth / intrapartum / intra-partum / postpartum / post-partum / puerperal / puerperium / parturition / “expectant mother” / “expectant mothers”) **&** ((mortalit* / fatalit* / death*) **/** (morbidit* / “pregnancy complication” / “obstetric complication” / “obstetric labor complication” / “obstetric labour complication” / “adverse pregnancy outcome” / “postpartum haemorrhage” / “postpartum hemorrhage” / “obstetric haemorrhage” / “obstetric hemorrhage” / hemorrhage / “vaginal bleeding” / “antepartum haemorrhage” / “antepartum hemorrhage” / dystocia / “obstructed labour” / “obstructed labor” / “prolonged labour” / “prolonged labor” / “retained placenta” / “pregnancy induced hypertension” / hellp / eclampsia / preeclampsia / “pre-eclampsia” / “gestational diabetes” / “abruptio placent*” / “placental abruption” / “placenta previa” / "placenta praevia" / “ruptured uterus” / sepsis / septic / septicemia / septicemic / endometritis / “puerperal infection” / “near miss” / “near-miss” / “caesarean section” / c-section / “caesarian section” / “cesarean section” / anaemia / anemia) **/** (“CD4 lymphocyte count” / “CD4 count” / “HIV disease progression” / “HIV severity” / “aids defining” ***/ “***AIDS-related opportunistic Infections” / “kaposi's sarcoma” / lymphoma / “wasting syndrome” / cachexia / ***“***pneumocystis carinii” / tuberculosis / tb / “symptomatic HIV” / “opportunistic infection*”) / (seroconversion / incidence))

**AFRICAN INDEX MEDICUS SEARCH STRATEGY**

**HIV terms:**

HIV, human immunodeficiency virus, AIDS, acquired immunodeficiency syndrome

**Maternal/pregnancy terms:**

Maternal, Pregnancy, childbirth, intrapartum, intra-partum, postpartum, post-partum, puerperal, puerperium, parturition

**Searches conducted:**

1. Maternal HIV
2. Pregnancy HIV
3. Puerperium HIV
4. Maternal human immunodeficiency virus
5. Maternal AIDS
6. Pregnancy AIDS
7. Maternal acquired immunodeficiency syndrome
8. Pregnancy acquired immunodeficiency syndrome
9. Puerperium acquired immunodeficiency syndrome
